# Photochemical Control of Perovskite Crystal Formation at Room Temperature

**DOI:** 10.1002/advs.202522760

**Published:** 2025-11-29

**Authors:** Magdalena Breitwieser, Lukas E. Lehner, Julius Feigl, Lukas M. Rescher, Julia Felicitas Schwarz, Felix Mayr, Munise Cobet, Bekele Hailegnaw, Christoph Putz, Clemens Schwarzinger, Markus Clark Scharber, Markus Himmelsbach, Bert Nickel, Stepan Demchyshyn, Martin Kaltenbrunner

**Affiliations:** ^1^ Division of Soft Matter Physics, Institute of Experimental Physics Johannes Kepler University Altenberger Str. 69 Linz 4040 Austria; ^2^ Soft Materials Lab, Linz Institute of Technology Johannes Kepler University Altenberger Str. 69 Linz 4040 Austria; ^3^ Soft Condensed Matter Group, Faculty of Physics Ludwig‐Maximilian University Geschwister‐Scholl‐Platz 1 80539 Munich Germany; ^4^ Institute of Chemical Technology of Organic Materials Johannes Kepler University Altenberger Str. 69 Linz 4040 Austria; ^5^ Linz Institute for Organic Solar Cells (LIOS) and Institute for Physical Chemistry Johannes Kepler University Altenberger Str. 69 Linz 4040 Austria; ^6^ Institute of Analytical and General Chemistry Johannes Kepler University Altenberger Str. 69 Linz 4040 Austria

**Keywords:** 2D, crystallization, patterning, perovskites, photochemical processing, room temperature, UV light

## Abstract

Lead halide perovskites combine outstanding optoelectronic performance with low‐cost and scalable manufacturing routes. However, their commercial success remains limited by a still‐evolving understanding of crystallization dynamics, a lack of sustainable, material‐informed processing techniques, and persistent challenges in fabricating blue‐emitting and 2D compositions with sterically‐impeded cations. Here, the interaction of light with organic–inorganic perovskite precursor solutions is uncovered by presenting a photochemically‐assisted crystallization control technique (PACCT). This low‐energy method leverages UV illumination to modulate reaction kinetics, allowing controlled crystal formation in both thin films and flexible perovskite‐polymer composites under ambient atmosphere at room temperature. The treatment's influence on the crystallization process either impedes (3D perovskites) or promotes (2D perovskites) crystal growth. In the latter case, this facilitates the growth of perovskites that can otherwise be challenging using thermal approaches, enabling a facile pathway toward blue emission and thin films with a photoluminescence stability exceeding 1000 h at 80 % relative humidity. For both reaction pathways, mechanistic descriptions are identified based on the UV‐induced deprotonation of the organic cation. Together, these results introduce a novel strategy for perovskite fabrication and provide new insights into their crystallization dynamics, offering a versatile and scalable route for advancing next‐generation optoelectronic materials with a reduced energy footprint.

## Graphical abstract

The authors demonstrate how ultraviolet (UV) light can enable the crystallization of two‐dimensional (2D) perovskite crystals that are otherwise difficult to grow using conventional thermal methods. These findings are supplemented with a detailed mechanistic description for the interaction of the UV irradiation with the perovskite precursor components.

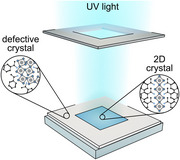

## 1 Introduction

Developing processing techniques that combine cheap and facile fabrication with a low energy footprint is critical for enabling the next generation of optoelectronic applications that are both economically viable and more sustainable. Solution‐processable organic–inorganic perovskites have emerged as promising candidates in this regard due to their highly adjustable chemical and physical properties, facilitating a wide range of processing techniques and material characteristics. In particular, their tunable bandgap,^[^ ^1^, ^2^ ^]^ narrowband and high‐purity emission,^[^ ^3^, ^4^ ^]^ and high defect tolerance,^[^ ^5^, ^6^ ^]^ make perovskite nanocrystals (PNCs) and thin films suitable for integration into a wide array of applications ranging from color‐converters,^[^ ^7^, ^8^ ^]^ and lasers^[^ ^9^, ^10^, ^11^ ^]^ to light emitting diodes^[^ ^12^, ^13^, ^14^, ^15^, ^16^, ^17^ ^]^ and solar cells.^[^ ^18^, ^19^, ^20^, ^21^, ^22^, ^23^ ^]^ However, their commercialization is still limited by an incomplete understanding of their crystallization dynamics, as well as hurdles when transitioning to scalable, energy‐efficient processing techniques. Further, facilitating blue emission in perovskites remains challenging despite significant progress in red and green emitting structures.^[^ ^24^, ^25^ ^]^ Current strategies for producing blue‐emitting perovskites primarily involve either reducing the nanoparticle size to induce quantum confinement,^[^ ^26^, ^27^ ^]^ or altering the bandgap via halide engineering.^[^ ^28^ ^]^ However, small, strongly confined PNCs suffer from a high probability of defect formation due to their large specific surface area. Meanwhile, mixed halide perovskites are prone to phase segregation, resulting in unstable emission spectra.^[^ ^29^ ^]^

Beyond size reduction, quantum confinement can also be realized using large organic cations (A) to for 2D perovskites with the general crystal structure A_2_BX_4_ (B is a divalent metal cation and X a halide anion). They consist of [PbX_6_]^4−^ octahedral sheets separated by the bulky A‐site cations, confining the charge carriers and modifying their electronic and optical properties. In addition, the large organic spacers can also enhance the material's hydrophobicity^[^ ^30^ ^]^ and mechanical resilience,^[^ ^31^ ^]^ suppress ion migration,^[^ ^32^ ^]^ or introduce chirality.^[^ ^33^ ^]^ However, the increased steric hindrance of these bulkier molecules results in higher formation energies.^[^ ^31^, ^34^ ^]^ This can cause phase impurities and defects in the devices, resulting in multi‐ or broadband emission,^[^ ^35^, ^36^, ^37^ ^]^ thus negatively impacting color purity and photoluminescence (PL) intensity. Employing chiral molecules like methylbenzylammonium (MBA) further exacerbates this issue, as the additional steric effects can impede proper 2D structure formation.^[^ ^36^ ^]^ Indeed, when using Br‐based perovskites, which have a higher formation energy compared to their I‐based counterparts,^[^ ^38^ ^]^ reports indicate that purely‐2D MBA_2_PbBr_4_ perovskites tend to have poor material properties and even PL signals below the detection limit.^[^ ^39^, ^40^, ^41^ ^]^ Therefore, given the rise in interest regarding chiral perovskites for spintronic applications,^[^ ^42^ ^]^ as well as in 2D perovskites more generally for light emission and photovoltaics,^[^ ^22^, ^43^ ^]^ improving and controlling their crystallization pathways is critical to obtain higher‐quality materials.

Crystallization in perovskites can be controlled through a variety of methods, including thermal management,^[^ ^44^, ^45^, ^46^ ^]^ additive^[^ ^47^, ^48^, ^49^, ^50^ ^]^ and solvent engineering,^[^ ^51^, ^52^, ^53^ ^]^ interface engineering,^[^ ^54^, ^55^, ^56^ ^]^ as well as light‐assisted approaches.^[^ ^57^, ^58^, ^59^, ^60^, ^61^ ^]^ The latter stands out as minimally invasive, easily adaptable, and compatible with existing scalable fabrication techniques. Given the sensitivity of the materials to light, previous work has explored the impact of illuminating the perovskite precursors at different stages of the fabrication procedure, particularly during crystallization.^[^ ^62^ ^]^ Some results indicate that illumination with UV or AM1.5 (air mass 1.5, simulated solar spectrum) positively impacts the perovskite formation,^[^ ^58^, ^59^, ^60^, ^61^ ^]^ while others clearly demonstrate a negative effect of similar treatments.^[^ ^63^, ^64^, ^65^ ^]^ Despite these varied observations, a comprehensive mechanistic explanation for how light affects various perovskite compositions has yet to be established.

Here, we investigate the role of UV illumination in modulating reaction kinetics in organic–inorganic hybrid lead halide perovskite precursor solutions and its influence on crystallization dynamics. Based on these findings, we demonstrate a novel photochemically‐assisted crystallization control technique (PACCT) that utilizes UV light to support perovskite formation under ambient conditions. This versatile method is effective across thin films, in situ PNCs embedded in polymer matrices, and a range of cation compositions. Notably, PACCT provides a facile, room temperature approach to preparing narrow‐band, blue‐emitting, low‐dimensional perovskite‐polymer composites as well as 2D perovskite thin films with PL stability exceeding 1000 h under 80% relative humidity (RH). Furthermore, by not relying on heating to form the samples, we estimate that this rapid UV light processing at ambient conditions can reduce the energy demand of 2D perovskite fabrication by about one order of magnitude compared to conventional thermal methods.

The underlying UV‐induced reaction mechanism is investigated using representative lead bromide perovskite compositions containing large MBA or small MA organic A‐site cations. This analysis reveals a common initial reaction with divergent kinetics that either promotes or suppresses crystallization. UV‐induced deprotonation of the ammonium group in A‐site cations results in amine formation. While the volatile methylamine rapidly evaporates, suppressing MAPbBr_3_ formation, the larger deprotonated MBA remains in solution. Critically, the changes to the precursor solution's colloidal structure caused by the illumination allow MBA to overcome steric hindrances, yielding perovskites of improved quality, which are otherwise challenging to achieve using conventional heat‐based fabrication approaches. This results in PL signals more than two orders of magnitude greater compared to samples fabricated via conventional annealing.

Together, these findings provide a new insight into the role of light in lead halide perovskite formation. Notably, besides facilitating the crystallization of perovskite systems that are otherwise challenging to grow into high‐quality materials using thermal methods, PACCT also enables both positive and negative direct patterning of perovskites at room temperature without introducing a photoresist or high‐power lasers. As a result, reduced energy consumption is combined with low cost and compatibility with existing large‐scale and high‐resolution fabrication techniques, while offering a novel path for developing advanced optoelectronic applications.

## 2 Results and Discussion

**Figure 1:**
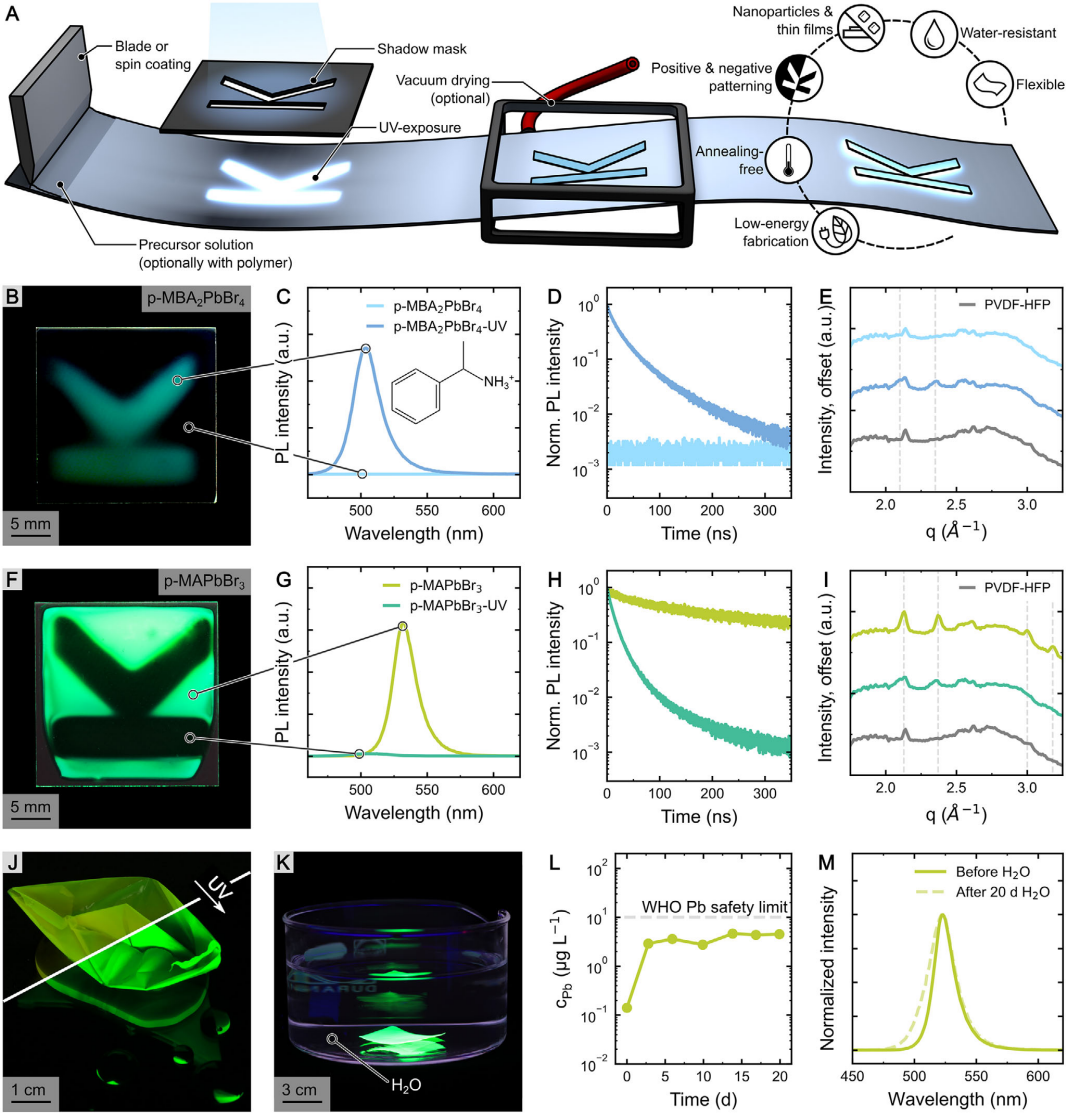
UV exposure of perovskites. A) Illustration of the sample preparation process starting with film deposition, followed by UV exposure (254 nm) and optionally completed with low vacuum drying. B) Photograph of a blade‐coated MBA_2_PbBr_4_‐PVDF‐HFP composite film (p‐MBA_2_PbBr_4_) under UV illumination. The luminescent K‐shaped region marks the area exposed to UV during fabrication. C) Photoluminescence (PL) and D) normalized time‐resolved photoluminescence (TRPL) of the same films demonstrating increased PL intensity and longer carrier lifetimes in UV‐exposed samples. E) Wide‐angle X‐ray scattering (WAXS) diffraction pattern of pristine and UV‐exposed p‐MBA_2_PbBr_4_. Grey dashed lines mark the peaks that appear during the UV treatment. F) Photograph of a MAPbBr_3_‐PVDF‐HFP composite film (p‐MAPbBr_3_) under UV illumination. The dark K‐shaped part marks the area exposed to UV during sample fabrication. G) PL and (H) normalized TRPL indicating reduced PL intensity and carrier lifetimes in UV‐treated samples. I) WAXS diffraction pattern of pristine and UV‐exposed p‐MAPbBr_3_. Grey dashed lines designate the peaks that degrade due to the UV exposure. MAPbBr_3_ polymer composite film peeled off and (J) folded to showcase the flexibility and (K) water‐repellent properties while sustaining bright PL. L) Pb leakage from a peeled‐off MAPbBr_3_ PVDF‐HFP composite film submerged in water for 20 days. The gray dashed line refers to 10 µg L^−1^, the maximally allowed Pb concentration in drinking water defined by the World Health Organization (WHO). M) Normalized PL spectra of MAPbBr_3_ polymer composite films before and after 20 days of full submersion in water.

To investigate the effect of UV irradiation on the perovskite formation, the PNCs were grown in situ inside a poly(vinylidene fluoride‐co‐hexafluoropropylene) (PVDF‐HFP) polymer matrix according to previous literature.^[^ ^56^ ^]^ After blade or spin coating, the still‐wet perovskite precursor solution is illuminated with low‐power UV light (254 nm) for 7 min (**Figure** **1**A). A subsequent (optional) exposure to a low‐pressure environment to control the solvent evaporation rate, followed by a second 7 min long UV illumination, can be implemented to accelerate the drying process and further enhance the effect of PACCT (Figure S1, Supporting Information). After the polymer has dried, the samples can then be left for minutes to hours in ambient conditions, during which the PNCs start to form. This temporal separation of polymer and PNC formation allows the facile UV‐treatment of the precursor components before they have fully crystallized.

When carrying out this procedure using the 2D MBA_2_PbBr_4_ perovskite system inside a polymer matrix (hereafter labeled as p‐MBA_2_PbBr_4_) without UV illumination, no PL is observed, indicating that no PNCs form. Only upon exposing the samples to UV (labeled as p‐MBA_2_PbBr_4_‐UV), does a strong PL signal emerge (Figure 1B–D), with a peak position at 502 ± 3 nm for blade‐coated and 496 ± 3 nm for spin‐coated samples. Upon optimization of the MBABr:PbBr_2_ ratio, this results in a photoluminescence quantum yield (PLQY) of up to 28% (Table S2, Supporting Information), while the PLQY of non UV‐treated samples was too low to measure. Note that this is likely an underestimate of the PLQY as the samples have a very low optical density.

The formation of crystalline structures is further confirmed by additional peaks in the wide‐angle X‐ray scattering (WAXS) pattern (Figure 1E; Figure S2, Supporting Information) emerging exclusively in UV‐exposed films, indicating the UV‐induced growth of nanoparticles with an estimated average diameter of 42 nm, as determined by a Guinier analysis of the small‐angle X‐Ray scattering (SAXS) pattern of p‐MBA_2_PbBr_4_‐UV (Figure S3A,B, Supporting Information).

In contrast, when using a small cation precursor composition, leading to the formation of 3D MAPbBr_3_ nanocrystals embedded in a PVDF‐HFP matrix (p‐MAPbBr_3_), UV exposure results in the inverse effect, that is, PL quenching (Figure 1F). The UV‐treatment causes the PLQY of 22 ± 4% of the pristine films to be reduced and become too low to reasonably measure. This reduction in emission intensity is accompanied by a hypsochromic shift (Figure 1G) as well as a decreased PL decay time (Figure 1H). The blue shift indicates a reduction in PNC size, which is validated by a Guinier analysis of the p‐MAPbBr_3_ SAXS data estimating a reduction in diameter from 41 to 22 nm, consistent with previous reports (Figure S3C,D, Supporting Information).^[^ ^66^ ^]^ Meanwhile, the PL decay time implies the degradation of the PNCs as evidenced by an increase of non‐radiative recombination due to a higher trap state density.^[^ ^67^ ^]^ A similar conclusion can be drawn from the decrease in WAXS signal intensity and broadening of the diffraction peak (Figure 1I). These alterations indicate a reduction in the number of PNCs maintaining a similar crystal structure, a decrease in overall crystal size, and an increase in lattice strain with more defects as a result of the UV‐induced degradation of the perovskite.^[^ ^68^ ^]^

During UV‐exposure, the UV‐lamp reaches a temperature of ≈40 °C, causing the samples to heat to ≈30 °C (Figure S4A,B, Supporting Information). Therefore, to determine whether the observed changes in crystallization are induced by UV illumination rather than radiative heat introduced by the lamp, both systems were subjected to temperatures up to 55 °C instead of UV exposure, while keeping all other conditions identical (Figure S4C,D, Supporting Information). In p‐MBA_2_PbBr_4_, heating does not induce PL emission. Heat exposure of p‐MAPbBr_3_ reduces PL intensity, but only after temperatures beyond 45 °C and with little to no hypsochromic shift. Further analysis of WAXS data from heat‐treated p‐MAPbBr_3_ samples reveals distinct peak splitting, while for UV‐exposed samples the same peaks vanish instead (Figure S4E, Supporting Information). Combined with the altered and distinctly different sample appearance upon heating (Figure S4F, Supporting Information), this confirms that the effect of UV illumination is not caused by the heat transferred to the sample. Further, influencing the solvent evaporation by annealing the films at 100 °C for 10 min instead of vacuum or air drying does not yield luminescent films, likely due to the premature crystallization of the polymer preventing perovskite formation (Figure S5, Supporting Information). This separates PACCT from other state‐of‐the‐art perovskite crystallization methods, which rely on local heating via high‐power laser light,^[^ ^69^, ^70^ ^]^ as well as procedures utilizing direct exposure to elevated temperatures, such as thermal annealing or hot injection. Instead, it opens up the fabrication of perovskite systems that are otherwise inaccessible by heat‐based procedures. By avoiding elevated temperatures during sample preparation, we estimate that PACCT leads to a significantly lower energy consumption of the 2D perovskite fabrication process. This in turn increases its economic viability and sustainability due to decreased costs and greenhouse gas emissions (Figure S6 and Table S3, Supporting Information). In addition, once formed, the samples are resistant to high temperatures, with dark regions remaining non‐emissive and luminescent regions retaining their PL signal even after being exposed to 100 °C for 60 min (Figure S7, Supporting Information). Therefore, the samples maintain their patterned structures even at elevated operating temperatures.

Embedding PNCs in polymers like PVDF‐HFP yields flexible and hydrophobic films, which help to protect the perovskite from environmental stressors and are of particular interest when used in display backlights or LEDs.^[^ ^56^, ^71^ ^]^ Additionally, favorable interactions between the polymer ‐CF_2_‐ dipole and the perovskite A‐site ammonium group further contribute to improved performance.^[^ ^56^ ^]^ The impact of the UV radiation on the morphology of the polymer matrix was investigated by recording atomic force microscopy (AFM, Figure S8, Supporting Information) and scanning electron microscopy (SEM) (Figure S9, Supporting Information) images. In both instances, no significant differences in the pristine and UV‐treated samples can be observed, indicating that the short UV exposure does not substantially damage the PVDF‐HFP. Therefore, the beneficial properties resulting from the polymer, such as its high flexibility (Figure 1J) and water resistance (Figure 1K), do not deteriorate. Even after 20 days of full submersion in water, the Pb content stays well below the maximum allowed amount in drinking water (10 µg L^−1^) as defined by the World Health Organization (WHO)^[^ ^72^ ^]^ (Figure 1L). Furthermore, the PL profile of the MAPbBr_3_ PNCs, which are intrinsically highly sensitive to moisture, does not change significantly before and after long‐term water exposure, with only a small shoulder appearing at lower wavelengths without a shift in peak position. This can be attributed to minor amounts of the hydrate phases degrading the crystal structure^[^ ^73^, ^74^ ^]^ (Figure 1M). Similarly, the p‐MBA_2_PbBr_4_‐UV system also exhibits no significant degradation after prolonged immersion in water (Figure S10, Supporting Information).

**Figure 2:**
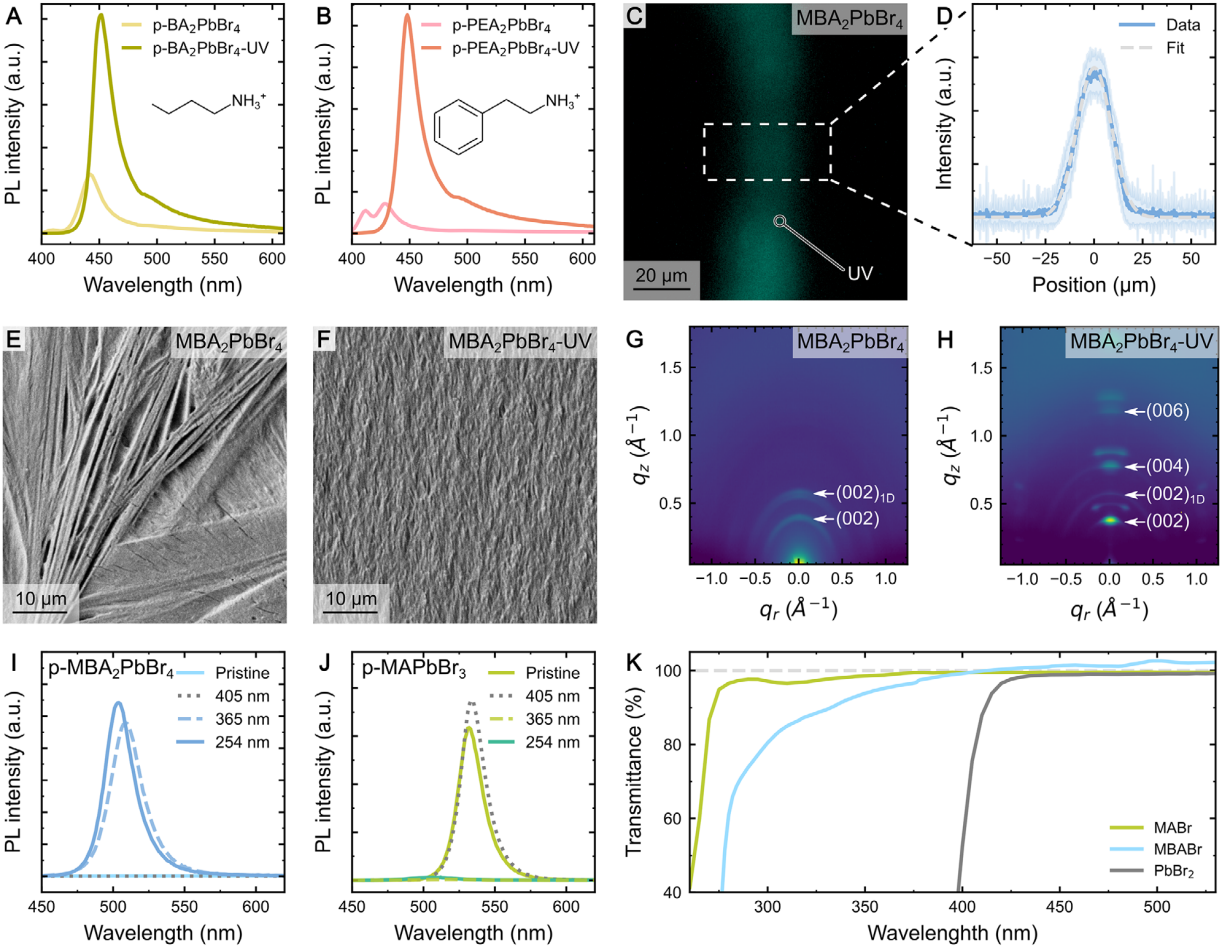
Applications and analysis of PACCT. A) PL spectra of BA_2_PbBr_4_ and B) PEA_2_PbBr_4_ polymer composite films demonstrating the increase in PL intensity due to the UV treatment. C) Micrograph of a patterned polymer‐free MBA_2_PbBr_4_ film and (D) spatial emission intensity distribution extracted from the image. The band represents the standard deviation as taken from averaging along the vertical direction inside the area indicated by dashed borders. E) SEM images of pristine (no UV) and (F) UV‐illuminated polymer‐free MBA_2_PbBr_4_ films. G) GIWAXS images of pristine and (H) UV‐exposed polymer‐free MBA_2_PbBr_4_ films reveal the emergence of a pronounced crystal structure in UV‐treated samples. Indices in brackets correspond to the facets of the 2D or 1D perovskite phases. I) PL spectra of MBA_2_PbBr_4_ and (J) MAPbBr_3_ PVDF‐HFP composite films, comparing pristine samples to those exposed to various wavelengths of light during fabrication. In both instances, only wavelengths below 405 lead to the patterning effect. (K) Transmittance spectra of selected perovskite precursor components dissolved in DMF.

The versatility of PACCT is further demonstrated by its application to two additional 2D perovskite compositions (**Figure** **2**A,B). UV exposure of precursor solutions containing the commonly employed large cations butylammonium (BA) or phenethylammonium (PEA) to form p‐BA_2_PbBr_4_ and p‐PEA_2_PbBr_4_ results in both systems exhibiting enhanced PL intensities. With their peak emission at λ_peak_(p‐BA_2_PbBr_4_‐UV) = 453 ± 4 nm (N = 4) and λ_peak_(p‐PEA_2_PbBr_4_‐UV) = 448 ± 1 nm (N = 4), these compositions achieve blue emission, in general agreement with previous single‐crystal emission studies.^[^ ^75^, ^76^ ^]^ Interestingly, the additional short‐wavelength PL peaks, attributed to surface states in both BA_2_PbBr_4_ and PEA_2_PbBr_4_, disappear after UV exposure. Meanwhile, the primary emission peak, associated with bulk states, exhibits both a red shift and an increase in PL intensity. This provides further evidence of a decreased surface‐area‐to‐volume ratio and, thus, larger crystals due to the UV treatment in the case of 2D perovskites. Similar effects have been observed in single crystals upon annealing at 120 °C and were attributed to improved packing of the A‐site spacer cation, reduced lattice distortion, and the passivation of surface states.^[^ ^76^ ^]^ UV irradiation thus provides a facile route of creating bright 2D perovskite structures at room temperature, showing great promise for future applications, especially concerning the presently challenging fabrication of blue light‐emitting structures.^[^ ^77^ ^]^

We further examined the process of UV‐induced crystallization by preparing polymer‐free MBA_2_PbBr_4_ thin films. Both films with and without the polymer show a comparable PL response (Figure S11A,B, Supporting Information), provided the latter was exposed to ambient humidity. Notably, the MBA_2_PbBr_4_‐UV films also demonstrate a strong PL response, while analogous films prepared using annealing at 130 °C instead show no PL signal (Figure S11C, Supporting Information). Once the crystals have formed, the unencapsulated, polymer‐free thin films showed excellent PL stability during prolonged exposure to elevated humidity levels of 80 % RH over more than 1000 h (Figure S12, Supporting Information). This demonstrates that even in the absence of the encapsulating polymer, PACCT does not diminish the stability typical for 2D perovskites, as the hydrophobic spacer cations limit moisture ingress, suppress ion migration, and passivate defects.^[^ ^78^ ^]^

Further patterning using a shadow mask allows the formation of simple structures at a resolution of down to 21 µm, as estimated by the full width at half maximum (Figure 2C,D). Although these proof‐of‐principle results are still modest when compared to state‐of‐the‐art lithography,^[^ ^79^ ^]^ they highlight the future potential of PACCT when translated to advanced semiconductor fabrication environments. Especially the advantages of the PACCT approach, such as fewer processing steps, minimized additional chemical agents, and elimination of heat exposure, can be exploited for future research in the fields of photovoltaics, light‐emitting structures, and sensing applications.

Studying these MBA_2_PbBr_4_ films via SEM reveals a stark difference between the surfaces of pristine (Figure 2E) and UV‐exposed (Figure 2F) samples. While the former shows a non‐uniform morphology broken up by micro‐cracks, the latter presents a more homogeneous and superstructure‐free surface. Further SEM analysis of annealed (40 °C) instead of vacuum‐dried pristine MBA_2_PbBr_4_ films shows a similarly uneven morphology, indicating the improved surface quality is indeed caused by the UV‐light rather than an increased solvent evaporation rate caused by the slight heating of the UV lamp (Figure S13A, Supporting Information). In case of MAPbBr_3_, the polymer‐free pristine film shows a continuous morphology (Figure S13B, Supporting Information). UV‐exposure, however, causes the formation of individual, broken‐up domains instead of forming a continuous thin film, indicating severe degradation (Figure S13C, Supporting Information). X‐ray photoelectron spectroscopy (XPS) measurements of the same films reveal that the UV‐treatment severely diminishes the N:Pb ratio in the sample, from ≈3:1 to 1.8:1, indicating a loss of the organic species due to radiation damage (Figure S14A and Table S4, Supporting Information). Conversely, the composition of MBA_2_PbBr_4_ does not significantly change despite being subjected to the same high‐energy irradiation, suggesting that the large molecule remains mostly unaffected, likely due to its bigger size and therefore being less volatile (Figure S14B and Table S4, Supporting Information).

Grazing‐incidence WAXS (GIWAXS) images of both systems (Figure 2G,H) reveal the development of a pronounced crystal structure upon UV irradiation, as evidenced by the appearance of several Bragg peaks. Notably, the emergence of the (002*l*) peaks with an interlayer spacing of 16.2 Å, as well as the series of peaks at *q*_r_ = ± 1.1 Å^−1^, indicates improved long‐range order and a preferentially oriented 2D phase in the UV‐treated samples.^[^ ^80^, ^81^ ^]^ While some remnants of the 1D phase indicated by the (002*l*)_1D_ peak at *q* = 0.58 Å^−1^ remain in films exposed to UV,^[^ ^82^ ^]^ they are much less pronounced compared to the control samples. All reflections closely resemble those observed in conventionally processed MBA‐based films as previously reported in the literature.^[^ ^36^, ^81^, ^82^, ^83^, ^84^, ^85^ ^]^

To further elucidate the effect of UV exposure, we studied the impact of the used wavelength on the crystallization process of p‐MAPbBr_3_ and p‐MBA_2_PbBr_4_ films (Figure 2I,J). Both PL quenching in the MA‐based system and the onset of emission in the MBA‐based perovskite emerge only upon exposure to wavelengths below 405 nm, with strong effects being observed at 365 nm. This result is consistent with the onset of absorbance for both MABr and MBABr (Figure 2K), hinting at the importance of the organic cations to the underlying mechanism of PACCT.

**Figure 3:**
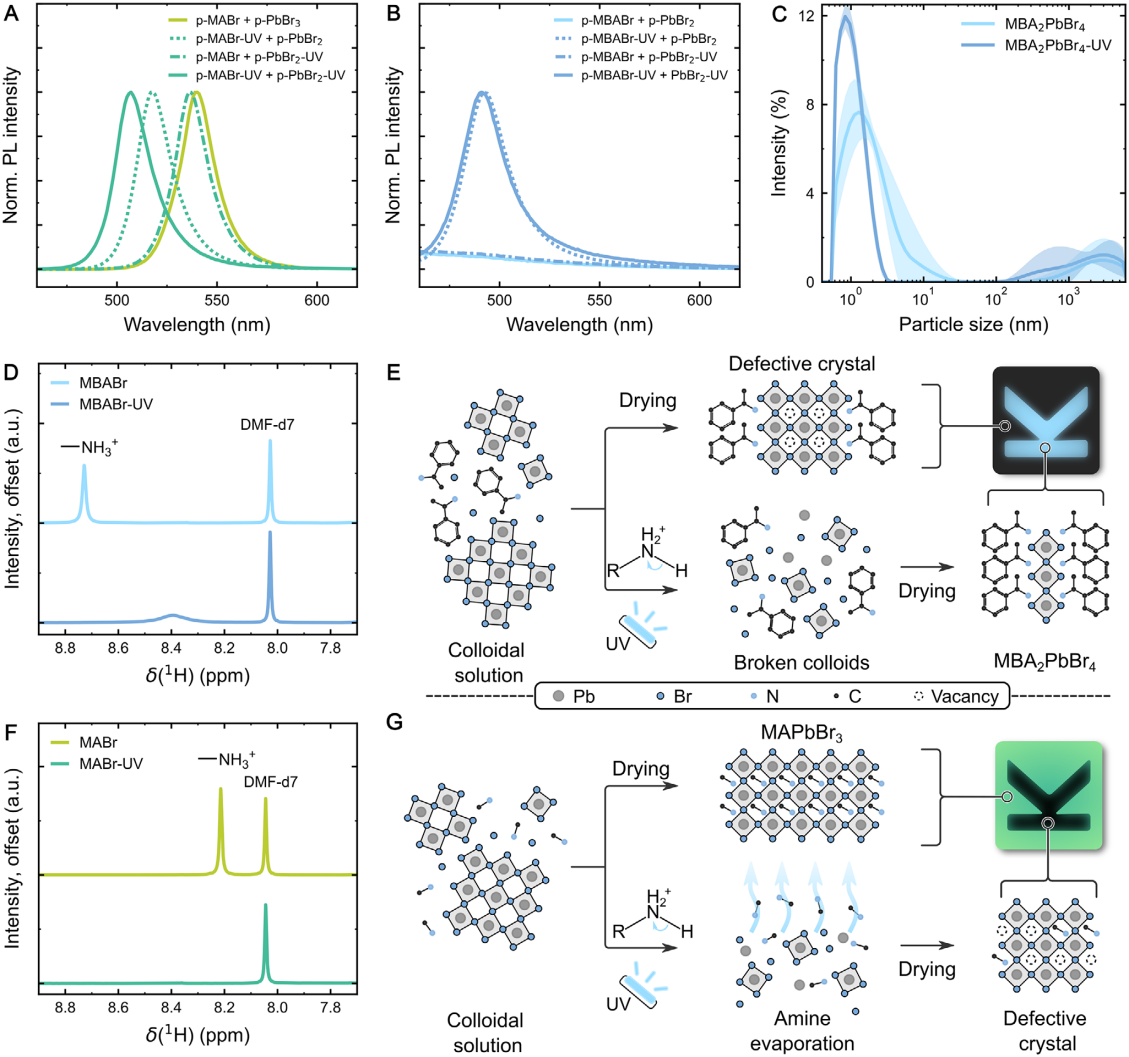
Mechanism investigation. A) Normalized PL spectra of MAPbBr_3_ and (B) MBA_2_PbBr_4_ polymer composite films, with individual components of the precursor solution being selectively exposed to UV during fabrication. C) DLS measurements of MBA_2_PbBr_4_ precursor solutions before and after UV treatment. Bands indicate the standard deviation from 15 measurements. D) NMR spectra of pristine and UV‐exposed MBABr displaying the change of the ammonium peak after UV irradiation. E) Schematic illustrating the UV‐assisted formation of luminescent MBA_2_PbBr_4_ crystals. The colloidal nature of the precursor solution leads to the formation of defective crystals upon drying, as the size of the MBA cations prevents their diffusion into the lead bromide complexes. UV‐induced deprotonation results in the dissociation of the colloidal structures, allowing the formation of luminescent crystals. F) NMR spectra of pristine and UV‐exposed MABr showing the vanishing of the ammonium peak resulting from the UV treatment. G) Illustration of UV‐assisted PL quenching of MAPbBr_3_. Dissolving the precursor components results in a colloidal solution. Upon drying, perovskite crystals with a low defect density are formed. UV exposure causes deprotonation of the MA^+^ cations, leading to evaporation of the resulting methylamine. This process reduces the number of available cations, causing defective crystal formation.

Additional insights into the role of the individual precursor components were gained by selectively irradiating them with UV light. Consequently, PVDF‐HFP precursor solutions containing only ABr (p‐ABr) or PbBr_2_ (p‐PbBr_2_) were UV‐exposed and processed as previously described. These films were then peeled off, re‐dissolved, and mixed together to form the final perovskite film (Figure S15, Supporting Information). Normalized PL spectra of films containing p‐MABr and selectively UV illuminated p‐PbBr_2_ exhibited minimal spectral shift compared to the reference, samples prepared with pristine p‐MABr and p‐PbBr_2_ (**Figure** **3**A). In contrast, a strong blue shift was observed in samples prepared with UV‐illuminated p‐MABr. Similarly, films derived from p‐MBABr and selectively UV‐exposed p‐PbBr_2_ solutions showed no discernible PL emission, whereas those prepared with UV‐illuminated p‐MBABr exhibit a strong PL response, closely aligning with a reference film containing solely UV‐treated components, p‐MBABr as well as p‐PbBr_2_. Next, dynamic light scattering (DLS) of MBABr and PbBr_2_ dissolved in DMF was measured to determine the impact of the UV light on the precursor solution (Figure 3C). In pristine solutions, we observe the smaller colloids in the bimodal distribution to have a size of up to 20 nm. Upon UV‐treatment, however, their size is significantly reduced to a maximum of 2–3 nm. This indicates that the UV illumination likely causes the Pb‐Br bond to expand, ultimately leading to the dissociation of the [PbBr_6_]^4−^ colloids. It is important to note that the interpretation of DLS measurements, in particular regarding the larger particles/aggregates (>100 nm), has been debated in the literature.^[^ ^86^, ^87^, ^88^ ^]^ Due to their much stronger scattering, large structures will disproportionately affect the scattered intensity while only being present in negligible quantities. Nonetheless, the small‐particle regime of the bimodal distribution relevant for this work is reliably characterized using DLS, as demonstrated previously.^[^ ^86^ ^]^

Investigating this further, gas chromatography‐mass spectrometry (GC‐MS) of a redissolved and UV‐treated polymer‐free MBA_2_PbBr_4_ thin film identifies methlybenzylamine as well as 2‐phenylpropanal in the solution (Figure S16, Supporting Information). However, GC‐MS cannot determine the amount of positively charged MBA left in the sample. Therefore, to establish the chemical composition of this precursor solution in more detail, nuclear magnetic resonance (NMR) spectra are measured (Figure 3D). Here, a drastic decrease of the peak marking the ammonium group (‐NH_3_^+^) is observed when comparing the spectra of pristine and UV‐exposed MBABr (Figure 3D). The shifting and broadening of the ammonium peak is indicative of a change in pH of the solution, as would be expected upon deprotonation and subsequent evaporation of HBr. Notably, no new peaks appear characteristic for 2‐phenylpropanal, indicating that the amount of newly formed methylbenzylamine far exceeds that of 2‐phenylpropanal (Figure S17, Supporting Information). Raman spectroscopy (RS) of pristine and UV‐treated polymer‐free MBABr also shows no discernable difference between the two spectra (Figure S18, Supporting Information). This further supports the conclusion that the deprotonation of methylbenzylammonium into methylbenzylamine is the predominant outcome of UV exposure in these samples.

Considering all of the previously discussed findings, we propose the following description of the formation of an ordered crystalline MBA_2_PbBr_4_ structure upon exposure to UV light (Figure 3E). Mixing the precursor components leads to the formation of a colloidal perovskite solution, as shown previously.^[^ ^89^, ^90^ ^]^ Upon removal of the solvent, the bulky methylbenzylammonium cation is sterically hindered from diffusing into the lead bromide colloidal complexes, leading to the formation of a defective crystal structure full of cation vacancies. Exposure to UV light, however, leads to the formation of uncharged methlybenzylamine, as described by the following reaction:

(1) $${\text{RNH}}_{3}^{+}+{\text{Br}}^{-}\overset{h\nu }{\to }{\text{RNH}}_{2}+\text{HBr}$$

The volatile HBr mostly evaporates, resulting in an irreversible reaction. Without a sufficient number of cations stabilizing the highly negatively charged colloid complexes, their structure becomes unstable and is prone to breaking apart. This affects the pre‐nucleation of the perovskites as it reduces the size of the colloidal lead bromide structures, facilitating the diffusion of the larger organic molecules into the complexes. Note that this is likely aided by the direct destruction of the colloids by the UV light, since the effect of UV light on PbBr_2_ can also be observed even without any organic cations in the system, as evidenced by the reduction in the absorbance peak at 280 nm corresponding to lead‐halide structures (Figure S19, Supporting Information).^[^ ^60^, ^91^ ^]^ The dissociated colloid components can then potentially further aid the deprotonation through a range of side reactions (Supplementary Note). Upon reprotonation of the methylbenzylamine via exposure to ambient moisture, excess water inside the polymer matrix, or residual HBr in the system, a crystalline structure with a drastically reduced defect density forms, explaining the emerging PL properties of the material.

Note that both BA and PEA‐based perovskites show a small PL signal, even without UV exposure, that becomes stronger upon illumination (Figure 2A,B). This is likely due to the fact that their alkyl chains are flexible enough to make the proper arrangement and diffusion of the molecules into the colloids during crystallization more feasible. However, as the molecules become more bulky and rigid, going from BA (long alkyl chain) to PEA (phenyl group with short chain) to MBA (benzyl with chiral methyl group), the likelihood of forming luminescent crystals decreases due to the larger required crystallization energies.^[^ ^31^ ^]^ Because of this, exposing MBA_2_PbBr_4_ to UV for up to 7 min during fabrication continuously increases the PL of the final film, whereas the less sterically‐impeded BA‐based crystals have a smaller energy barrier to overcome and therefore their PL signal already plateaus after ≈5 min of exposure (Figure S20A,B, Supporting Information). Therefore, the introduction of UV light then enables the precursor components to kinetically overcome this energy barrier to form perovskites with enhanced crystallinity.

For the MA‐based system, the UV treatment initially leads to an analogous deprotonation of the ammonium group, as indicated again by a drastically reduced peak in the NMR spectrum (Figure 3F). However, this process has the opposite effect on the MAPbBr_3_ crystal structure (Figure 3G): Due to their small size, in the absence of UV radiation, the MA cations easily diffuse into the lead bromide complexes, leading to the formation of comparably low defect MAPbBr_3_ crystals. Exposure to UV light, however, induces the formation of the highly volatile methylamine,^[^ ^92^ ^]^ which evaporates alongside the solvent during the drying process. This markedly reduces the number of available cations, causing the formation of a defective crystal structure full of cation vacancies, as well as overall smaller PNCs, explaining the PL quenching and PL shift. Previous reports have demonstrated that illumination can be beneficial by reducing the nucleation barrier during sequential deposition (where MAI diffuses into a pre‐formed PbI_2_ film), but detrimental in single‐step processes.^[^ ^62^ ^]^ These findings are consistent with our proposed mechanism, as the cation diffusion can be aided by UV light breaking up the lead‐halide framework. In contrast, since no diffusion through the PbI_2_ bulk is required in the single‐step deposition, the light‐induced degradation of the cations outweighs this positive effect and decreases film quality.

## 3 Conclusion

We have developed a novel photochemically‐assisted crystallization control technique (PACCT) that leverages UV illumination to modulate lead halide perovskite formation. By tuning the precursor chemistry, we demonstrated the ability to either promote or hinder crystallization, enabling both positive and negative lithographic patterning. This method supports the processing of thin films and in situ growth of PNCs within polymer matrices, accommodates a wide range of organic cations with varying sizes and steric profiles (e.g., MBA, PEA, BA, and MA), and is applicable to both conventional 3D and blue‐emitting, low‐dimensional perovskite structures. Time‐resolved PL, GIWAXS, XPS, GC‐MS, and NMR analysis of MBA_2_PbBr_4_ and its constituents reveals that the UV‐treatment significantly suppresses defects in the film while enhancing its crystallinity without substantially degrading the organic cation. This results in a material that combines a PL intensity more than two orders of magnitude greater compared to their thermally‐processed counterparts, with the environmental resilience common in 2D perovskites.

In addition, we propose a kinetic mechanism of light‐precursor interaction supported by studies on MBA‐ and MA‐based lead bromide perovskites that explains the divergent effects of UV illumination. In the MBA‐based system, the crystallization is suppressed under the absence of light due to the steric hindrance of the bulky cation. UV exposure induces MBA ion deprotonation, forming neutral methylbenzylamine and disrupting colloidal lead bromide structures, thereby facilitating improved diffusion of the bulky molecules. Subsequent moisture‐assisted amine reprotonation allows for the formation of crystalline structures with reduced defect density. Conversely, MA‐derived perovskites readily crystallize under ambient conditions. However, UV illumination results in the formation of volatile methylamine upon deprotonation, which evaporates alongside the solvent. This disrupts the precursor stoichiometry, leading to perovskites with a high defect density and poor PL performance.

Combined, these results open new opportunities for fabricating advanced photonic structures, light‐emitting diodes, photovoltaics, and other optoelectronic devices with a significantly reduced energy footprint. Future work can focus on extending PACCT to perovskite compositions incorporating inorganic cations, halide and B‐site substitutions, as well as high‐resolution UV patterning to further broaden its applicability.

## 4 Experimental Section

### Precursor Solutions

Unless otherwise stated, the following steps were carried out in an inert N_2_ atmosphere (H_2_O < 0.1 ppm, O_2_ < 0.1 ppm).

### Perovskite‐Polymer Solutions

Before preparation of the solutions, BA and PEA salts were purified by first dissolving them in ethanol, followed by re‐precipitation using diethyl ether and subsequent freeze‐drying to remove residual solvents. The polymer‐perovskite precursor solution was prepared by mixing either MABr (14.32 mg, 0.128 mmol), MBABr (51.68 mg, 0.26 mmol), BA (40.05 mg, 0.26 mmol) or PEA (52.54 mg, 0.26 mmol) with PbBr_2_ (58.63 mg, 0.16 mmol), PVDF‐HFP (1.6825 g) as well as DMF (11 mL) and stirring at 60 °C for 6–8 h. For the PLQY‐optimization of p‐MBA_2_PbBr_4_ and p‐MAPbBr_3_, MBABr:PbBr_2_ ratios of 0.5:1, 0.75:1, 1:1, 1.25:1 and 1.6:1 or MABr:PbBr_2_ ratios of 0.6:1, 0.8:1, 1:1, 1.2:1, and 1.4:1 were prepared relative to 0.16 mmol PbBr_2_. Before deposition, the MAPbBr_3_ precursor solution was further diluted with a PVDF‐HFP solution (1.6825 g PVDF‐HFP dissolved in 11 mL DMF) to a weight ratio of 1:0.25.

For the experiments involving the selective UV illumination of the individual perovskite precursor components, a solution was prepared by mixing PVDF‐HFP (1.6825 g) and DMF (11 mL) together with either MABr (28.62 mg, 0.256 mmol) or MBABr (103.36 mg, 0.52 mmol) or PbBr_2_ (117.26 mg, 0.32 mmol). The solutions were then dissolved on a hotplate at 60 °C for ≈6 h and cooled down to room temperature before use.

### Non‐Polymer Perovskite Precursor Solutions

For samples not containing the polymer, perovskite precursor solutions were prepared by mixing MBABr (404.20 mg, 2 mmol) and PbBr_2_ (367.01 mg, 1 mmol) with DMF (2 mL). They were then left to stir overnight in an inert atmosphere at room temperature and stored in a closed glass vial in afore mentioned conditions until use.

### Polymer‐Nanoparticle Composite Film Preparation

1 sq. inch glass substrates were cleaned in Hellmanex solution (2 vol% in DI (deionized) water), DI water, acetone, and IPA for 15 min, respectively, inside an ultrasonic bath (FB15015, Fisherbrand, Canada). The substrates were then blow‐dried with N_2_ and fabricated by either blade‐coating 95 µL precursor solution at 2.5 mm s^−1^ velocity and a blade height of 2 mm (1 mm as measured from the substrate surface) in ambient conditions or spincoating 100 µL of precursor solution using a K.L.M. SCC‐200 spin coater at 1000 rpm for 30 s with an acceleration of 500 rpm s^−1^ in inert conditions.

### Pristine Films

Right after the deposition, the sample was placed inside a vacuum chamber for 7–15 min connected to a pump (ILMVAC GmbH membrane pump MPC 301 Z S2.3/2.5 m^3^ h^−1^) to remove most of the solvent at low pressure (30 mbar). Immediately after the film turned milky white, the vacuum was turned off, and the sample was kept in an ambient atmosphere. Final formation of nanoparticles took place in the ambient atmosphere.

### UV Exposed Films

After blade coating, the wet film was placed under a UV source (UVG‐54 Handheld UV Lamp, 6 W, 254 nm) for 7 min. As in the case of pristine films, the sample was placed inside a vacuum chamber and kept under low pressure (30 mbar) until the film fully dried (7–15 min). Immediately after vacuum drying, the film was once again exposed to UV light for 7 min. To determine the effects of different UV exposure times, the first exposure was varied from 1 to 7 min, and no second UV irradiation was performed. When varying the second exposure time, all samples were first subjected to 7 min of UV radiation during the first step. After this procedure, the nanoparticles formed within several minutes (in the case of MA‐based perovskites) to hours (in the case of MBA‐based perovskites). The samples were then stored in ambient conditions.

### Perovskite Films Without PVDF‐HFP

1 sq. inch glass substrates were sequentially cleaned in Hellmanex solution (2 vol% in DI (deionized) water), DI water, acetone, and IPA for 15 min, respectively, inside an ultrasonic bath (FB15015, Fisherbrand, Canada). Before use, they were dried under a pressurized N_2_ gas flow, and the surface of the substrates was O_2_ plasma treated inside a plasma oven (PE‐25, Plasma Etch Inc., U.S.A.) at maximum available power for 10 min.

The deposition of the perovskite films without PVDF‐HFP was conducted in an inert atmosphere. 100 µL of precursor solution was deposited using a K.L.M. SCC‐200 spin coater at 1000 rpm for 30 s with an acceleration of 500 rpm s^−1^. Afterwards, the samples were stored in ambient conditions.

### UV Exposed Films

After spin‐coating, the film was placed under a UV source (UVG‐54 Handheld UV Lamp, 6 W, 254 nm) for 7 min. The samples were then stored in ambient conditions. Depending on the humidity, crystalline structures formed after 1–2 days.

### Sample Characterization—Steady‐State Photoluminescence (PL)

All PL measurements were conducted using a Photon Technology International (PTI) spectrometer equipped with double monochromators on both the excitation and detection channels. An excitation wavelength of 405 nm and an illumination intensity of 0.4 mW cm^−2^ were used.

### Dynamic Light Scattering (DLS)

A 0.5 M solution of 2:1 MBABr:PbBr_2_ in DMF was filtered through a 0.2 µm PTFE filter to remove any dust. Before filtering, the filters were rinsed with pure DMF. DLS measurements were performed using a Zetasizer Nano ZSP (Malvern Panalytical, Germany) with an equilibration time of 120 s, a delay between measurements of 5 s, and a measurement angle of 173° (backscatter). The solutions were filtered and then left to rest for 60 min before being measured. Afterwards, the solutions were UV‐treated (254 nm) for 30 min and immediately measured again.

### Nuclear Magnetic Resonance (NMR) Spectroscopy

20 mM of either MABr or MBABr was dissolved alongside PbBr_2_ in DMF‐d7 at room temperature inside a N_2_‐filled glovebox. For samples exposed to UV, the liquid was filled into UV‐transparent quartz glass cuvettes (Hellma fluorescence cuvette, spectral range 200–2500 nm, pathlength 10 x 10 mm^2^, chamber volume 3500 µL) and illuminated using the UV lamp for 30 min (UVG‐54 Handheld UV Lamp, 6 W, 254 nm) under inert conditions. 1H NMR spectra (300 MHz) of the sample were recorded on a Bruker Avance 300 MHz spectrometer

### Grazing‐Incidence Wide‐Angle X‐Ray Scattering (GIWAXS)

GIWAXS measurements were performed in‐house with a Pilatus 100k from Dektris as a detector. A microfocusing X‐ray tube with an energy of 17.45 keV and a wavelength of 0.07107 nm (Mo K_α_) was used as the source. The sample‐detector‐distance (SDD) was between 0.14 m and 0.31 m, and the setup was calibrated with a known lanthanum hexaboride (LaB_6_) crystal powder filled in a capillary.

To differentiate between 3D and non‐3D phases, the 2D detector images were azimuthally integrated into the *q*‐*I* table with the Python module pyFAI, and the observed peaks were compared to existing literature.

## Conflict of Interest

The authors declare no conflict of interest.

## Author Contributions

M.B. and L.E.L. contributed equally to this work. M.B., S.D., and J.F. conceived the project. M.B., L.E.L., and S.D. conducted the preparation and fabrication of samples and the characterization of their PL. M.B. and S.D. conducted the investigations regarding different wavelengths and exposed different precursor components. M.B. and L.E.L. were responsible for analyzing all the data, as well as measuring SEM. L.E.L. performed the transmittance, resolution, and NMR experiments. L.M.R. and B.N. analyzed the samples using X‐ray scattering. C.S. and J.F.S. performed the Pb‐leakage tests. F.M. measured Raman spectra on liquid samples. M.C. performed the XPS measurement. B.H. conducted AFM measurements. L.E.L. and B.H. synthesized the organic salts. M.B., L.E.L., and C.P. designed the experimental setups. M.H. performed the GC‐MS measurements. M.B., F.M., and M.C.S. investigated the samples using time‐resolved PL. M.B., L.E.L., and M.C.S. performed PLQY measurements. M.B., L.E.L., S.D., C.P., and M.K. designed the figures and wrote the manuscript. All authors contributed to editing the manuscript. M.K., B.N., M.C.S., and C.S. supervised the research.

## Supporting information



Supporting Information

## Data Availability

The data that support the findings of this study are available from the corresponding author upon reasonable request.
