# Supplementary material for: Photochemical Control of Perovskite Crystal Formation at Room Temperature
Source: Adv Sci (Weinh). 2025 Nov 29;13(9):e22760. doi: 10.1002/advs.202522760 (PMC12903977; doi:10.1002/advs.202522760)
Supplement: Supplementary file 1 — Supporting Information [file ADVS-13-e22760-s001.pdf]

Supplementary Information

Photochemical Control of Perovskite Crystal Formation at Room Temperature

Magdalena Breitwieser[1, 2†], Lukas E. Lehner[1, 2†], Julius Feigl[1, 2], Lukas M. Rescher[3], Julia Felicitas Schwarz[4], Felix Mayr[5], Munise Cobet[5], Bekele Hailegnaw[1,2], Christoph Putz[1,2], Clemens Schwarzinger[4], Markus Clark Scharber[5], Markus Himmelsbach[6], Bert Nickel[3], Stepan Demchyshyn[1, 2], Martin Kaltenbrunner[1, 2*]

[†]These authors contributed equally to this work.

[1]Division of Soft Matter Physics, Institute of Experimental Physics, Johannes Kepler University, Altenberger Str. 69, 4040 Linz, Austria

[2]Soft Materials Lab, Linz Institute of Technology, Johannes Kepler University, Altenberger Str. 69, 4040 Linz, Austria

[3]Soft Condensed Matter Group, Faculty of Physics, Ludwig-Maximilian University, Geschwister-Scholl-Platz 1, Munich, Germany

[4]Institute of Chemical Technology of Organic Materials, Johannes Kepler University, Altenberger Str. 69, 4040 Linz, Austria

[5]Linz Institute for Organic Solar Cells (LIOS) and Institute for Physical Chemistry, Johannes Kepler University, Altenberger Str. 69, 4040 Linz, Austria

[6]Institute of Analytical and General Chemistry, Johannes Kepler University, Altenberger Str. 69, 4040 Linz, Austria

[*]Corresponding author. Email: martin.kaltenbrunner@jku.at

Materials

Unless otherwise specified, all chemicals were used as received without further purification and are listed in **table S1**.

Table S*1*: Chemicals. List of all the chemicals used, their supplier, and their country of origin.

Short name	Commercial name, purity	Supplier	Country
Acetone	Acetone, technical	VWR Chemicals	France
BABr	n-Butylammonium bromide	Sigma-Aldrich	Germany
DMF-d7	N,N-Dimethylformamide-d7, ≥ 99.5 at%D	Sigma Aldrich	Germany
DMF	N,N-dimethylformamide, 99.8 % anhydrous	Sigma-Aldrich	U.S.A.
Et2O	Diethyl ether	VWR Chemicals	Belgium
HBr	Hydrobromic acid, 48 wt%	Sigma-Aldrich	Germany
Hellmanex	Hellmanex III	Hellma Analytics	Germany
IPA	2-Propanol	VWR Chemicals	France
MABr	Methylammonium bromide, CH_3NH_3Br	Gretacell solar	Germany
MBAmine	(R)-(+)-α-methylbenzylamine, $C_8H_9NH_2$	Sigma-Aldrich	Germany
$PbBr_2$	Lead bromide, 99.9%	Sigma-Aldrich	Germany
PEABr	Phenetylammonium bromide, ≥ 98%	Sigma-Aldrich	Germany
PVDF-HFP	Poly(vinylidene fluoride-co-hexaflourpropyl-ene), Mw 40,000, Mn 130,000	Sigma-Aldrich	Germany

Methods

Synthesis of alpha-methylbenzylammonium bromide (MBABr)

MBA salts were synthesized according to previous work [1]. An equimolar amount of hydrobromic acid was slowly added to the alpha-methylbenzylamine under constant stirring inside an ice bath. The reaction mixture was diluted using ethanol (double the volume of HBr) and continued stirring for at least 2 h. Excess solvent was subsequently evaporated in a rotary evaporator (BUCHI Rotavapor R-114 with the water bath B-480). The powder was re-dissolved in small amounts of ethanol and re-precipitated and washed using diethyl ether. Afterwards, the powder was freeze-dried to remove residual solvents. Until use, the salt was stored under inert conditions inside a N_2 glovebox.

Polymer-nanoparticle composite film preparation

Heat exposed films: Immediately after blade coating, the wet film was placed on a hotplate in ambient conditions for 7 min. If the film had not completely dried during this step, it was then put into a vacuum chamber connected to a pump (ILMVAC GmbH membrane pump S2.3/2.5 m^3 h^{-1}) and kept under low pressure (30 mbar) until fully drying. Subsequently, the film was placed on a hotplate for 7 more min.

Exposure to different wavelengths: The procedure is analogous to the one described in the section "UV exposed films", with the exception of the light source. Samples illuminated with a wavelength of 405 nm were exposed to laser light of a Coherent OBIS 405LX. Films treated with a 365 nm wavelength were illuminated by a LED (Hönle UV technology, LED Spot 100, 365 nm, powered by a LED powerdrive 40)

Sample Characterization

Time-correlated single photon counting (TCSPC): Photoluminescence decay was measured using a pulsed supercontinuum white laser (SuperK EXTREME FIU-15, NKT Photonics) connected to a monochromator (LLTF contrast, Photon etc.). The signal was detected by a monochromator (DeltaNu DNS-300, Becker and Hickel GmbH) equipped with a photomultiplier-tube (PMC-100-1, grating: 600 l mm^{-1}, 500 nm blaze, slits: 3mm) at the wavelength of maximal PL as determined *via* steady-state PL. This TCSPC set-up had a full-width-at-half-maximum response function of around 230 ps.

Transmittance: 0.16 M of MABr, MBABr or $PbBr_2$ were dissolved in DMF. Transmittance spectra of the solutions were measured with a Perkin Elmer LAMBDA 1050 UV/Vis spectrometer and DMF as a reference. For the absorbance measurement of the diluted $PbBr_2$ solution, 10^{-4} M $PbBr_2$ was dissolved in DMF. After measuring the pristine solution, it was exposed to 254 nm UV illumination for 30 min inside a UV-transparent quartz glass vial and measured again. A longer treatment time was chosen to account for the much larger sample volume compared to the fabricated polymer composite films. The liquid was then left to rest for 60 min in the dark before being measured a third time.

Raman spectroscopy: Raman spectra were measured at an excitation wavelength of 1064 nm on a Bruker MultiRAM FT-Raman spectrometer, equipped with a liquid nitrogen-cooled Ge detector. Solutions of 1 M of MBABr in DMF were measured using a quartz glass cuvette (Hellma QS, 5 mm path length) with a mirrored rear wall. Raman spectra were recorded with a resolution of 2 cm^{-1} and by averaging 500 scans.

Patterning resolution: A polymer-free precursor solution of MBA_2PbBr_4 was spin-coated as described above. Before drying, the samples were placed under a shadow mask and exposed to UV light for 7 min (Hönle UV technology, LED Spot 100, 365 nm, powered by a LED powerdrive 40). Afterwards, the samples were vacuum-dried for 7 min and imaged using an optical microscope.

Gas chromatography-mass spectrometry (GC-MS): For GC-MS, 1 mg mL^{-1} MBABr was dissolved in DMF and the liquid was UV-treated (254 nm) for 40 min in a UV-transparent quartz-glass vial. In addition, a polymer-free MBA_2PbBr_4 film spin-coated and subsequently UV-treated for 7 min as described above. The finished films were then scraped off the glass substrate and redissolved in DMF (~ 1 mg mL^{-1}). Measurements were performed using an Agilent 6890 N Network GC with an Agilent 5975C Inert XL MSD mass spectrometric detector (Agilent Technologies, Santa Clara, USA) operated in scan mode (m/z 35-450) and an Optima 5 MS (30 m × 0.25 mm, 0.25 µm) GC-column (Macherey-Nagel, Düren, Germany). The temperature program was as follows: 120 °C isotherm for 1 min; up to 300° with 5 °C min^{-1}, hold time 3 min.

Moisture stability: The no-polymer MBA_2PbBr_4 films were fabricated as described above and subsequently exposed to atmospheric humidity inside a climate chamber (C-40/350, CTS Clima Temperatur Systeme GmbH) at 23 °C, 80 % RH. For PL measurements, the films were removed from the chamber and measured as described above.

Atomic force microscopy (AFM): Images were obtained using a “Bruker Innova” AFM.

Scanning electron microscopy (SEM): To characterize the surface, SEM images were recorded using a Zeiss 1540 XB CrossBeam scanning microscope at 5 keV.

Wide angle X-ray scattering (WAXS): WAXS measurements were performed at the German Electron Synchrotron (DESY - Deutsches Elektronen-Synchrotron) at a high energy beamline (P07). Pristine and UV irradiated films were peeled off, folded twice to form four layers. These samples were exposed to incident photons with an energy of E = 103.59 keV and a wavelength of λ = 0.11969 Å. The signal was detected by a VAREX detector.

Small-angle X-ray scattering (SAXS) & Guinier analysis: SAXS measurements were performed at DESY (beamline P62). The polymer-perovskite composite foils were folded three times (in total 8 layers) and placed on a sample holder for measuring them with an Eiger2 9M detector. A synchrotron source with an energy of E = 19.999 keV and a wavelength of λ = 0.61992 Å was used to perform the measurements.
To estimate the particle size, a Guinier fit was performed after subtracting the polymer background from the data. The radius of gyration (R_g) was determined by from Guinier’s approximation for the scattered intensity I at low scattering vectors q:

$$I(q) = I(0)e^{\frac{-q^2R_g^2}{3}}$$

From R_g, the diameter d of the nanocrystals is estimated by assuming a spherical particle:

$$d = 2\sqrt{\frac{5}{2}}R_g$$

Determination of Pb concentration in water *via* inductively coupled plasma mass spectroscopy (ICP-MS): For ICP-MS, samples were immersed in purified water (18.2 MOhm) and water

samples were extracted at regular intervals for up to 20 days. Subsequently, the lead concentration was analyzed without further dilution on a XSeries 2, Thermo Scientific ICP-MS instrument equipped with a MiraMist nebulizer, which was tuned for optimal sensitivity for lead while minimizing the oxide ratio. A Certipur multielement standard XXI was used to perform calibration. Four samples were analyzed and the one with the highest Pb-leakage was reported to indicate the worst-case scenario.

Photoluminescence Quantum Yield (PLQY): The absolute PLQY of spin-coated polymer-perovskite composites were measured using a Hamamatsu C9920-03 spectrometer equipped with an integrating sphere at an excitation wavelength of 405 nm.

X-Ray Photoelectron Spectroscopy (XPS): XPS measurements were performed using a ThetaProbe system (Thermo Scientific, UK). The specimens were probed with monochromated Al Kα X-Ray radiation (1486.6 eV) focused into a spot of 100 µm in diameter to ensure a good signal-to-noise ratio. Survey spectra were acquired using a pass energy of 200 eV and a binding energy (BE) step of 1 eV. To compensate for charges accumulating at the surface a dual flood gun was in use. The measured spectra were corrected with respect to the C1s peak of the adventitious carbon at 285.0 eV. Evaluation was performed using the Avantage software package provided by the device manufacturer.

Supplementary Note: Reaction Mechanism

Perovskite precursor solutions have been reported to form dispersed colloids rather than pure solutions [2]. The size of these colloids plays a vital role during the nucleation and subsequent crystallization of the perovskite crystals. Since the Pb-Br bond has a bond enthalpy of only 248 kJ mol^{-1} (2.58 eV per bond) [3], [4], UV illumination (with an energy of 4.88 eV) leads to an expansion of the Pb-Br bond, ultimately causing the partial decomposition of the $[PbBr_6]^{4-}$ octahedra [5]:

$$[\mathrm{PbBr_6}]^{4-} \xrightarrow{h\nu} \mathrm{Pb^0} + 4\mathrm{Br^-} + \mathrm{Br_2} \tag{1}$$

This allows the large MBA cation to more readily be incorporated into the inorganic framework. Upon further illumination, this may contribute to the deprotonation of the ammonium species as follows (R = CH_3- or $C_6H_5CH(CH_3)$-) [6]:

$$\mathrm{Br_2} \xrightarrow{h\nu} 2\mathrm{Br^\bullet} \tag{2}$$

$$2\mathrm{Br^-} + 2\mathrm{Br^\bullet} \xrightarrow{h\nu} 2\mathrm{Br_2^{\bullet-}} \tag{3}$$

$$2\mathrm{RNH_3^+} + 2\mathrm{Br_2^{\bullet-}} \xrightarrow{h\nu} 2\mathrm{RNH_2} + 2\mathrm{Br_2} + \mathrm{H_2} \tag{4}$$

Alternatively, the deprotonation can also occur through the formation of the tribromide anion:

$$\mathrm{Br^-} + \mathrm{Br_2} \overset{h\nu}{\rightleftharpoons} \mathrm{Br_3^-} \tag{5}$$

$$\mathrm{RNH_3^+} + \mathrm{Br_3^-} \rightarrow \mathrm{RNH_2} + \mathrm{Br_2} + \mathrm{HBr} \tag{6}$$

Note that reactions (5)-(6) do not necessarily require illumination but may also happen in the dark [6]. In both pathways, the Br_2 acts as a catalyst and can thus participate in further reaction cycles.

However, it should be noted that the deprotonation likely primarily occurs without other initiators like $PbBr_2$ or Br_2, as evidenced by the NMR measurements:

$$\mathrm{RNH_3^+} + \mathrm{Br^-} \xrightarrow{h\nu} \mathrm{RNH_2} + \mathrm{HBr} \tag{7}$$

Further, hydrohalic acids like HBr – as formed by reactions (6)-(7) – also aid in dissolving the existing lead polyhalide colloids ($[PbX_{2+n}]^{-n}$, $n \in \{1, \ldots, 4\}$) [7]. In case of MA-based perovskites (R = CH_3), the small, volatile amine can now evaporate, thus preventing the formation of defect-free nanoparticles. However, this does not happen in the case of MBA, since the cation is too large to evaporate at room temperatures.

Note that for the MBA_2PbBr_4 perovskites, the crystallization takes several hours to complete after illumination. During this time, the deprotonated RNH_2 is likely protonated again by either residual HBr left in the system or atmospheric water, since the equilibrium favors the ammonium formation in the absence of UV light.

Supplementary Figures

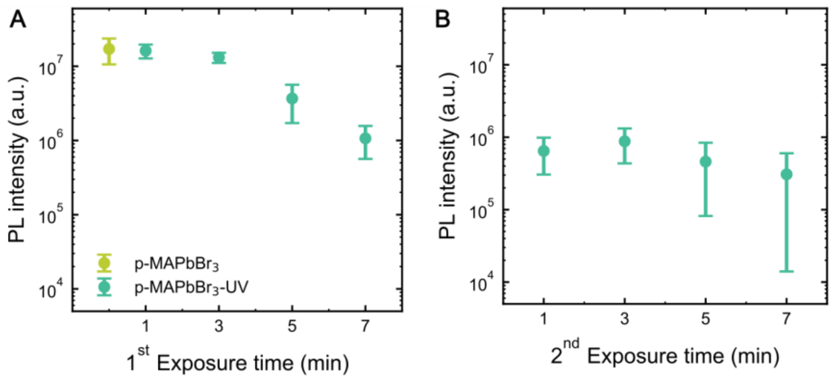

Figure S1. UV exposure time. (A) PL intensities of polymer-$MAPbBr_3$ composite films exposed to UV irradiation (wavelength λ = 254 nm) before and **(B)** after vacuum drying. The second exposure time followed a 7 min long first UV exposure.The error bars represent 3 samples.

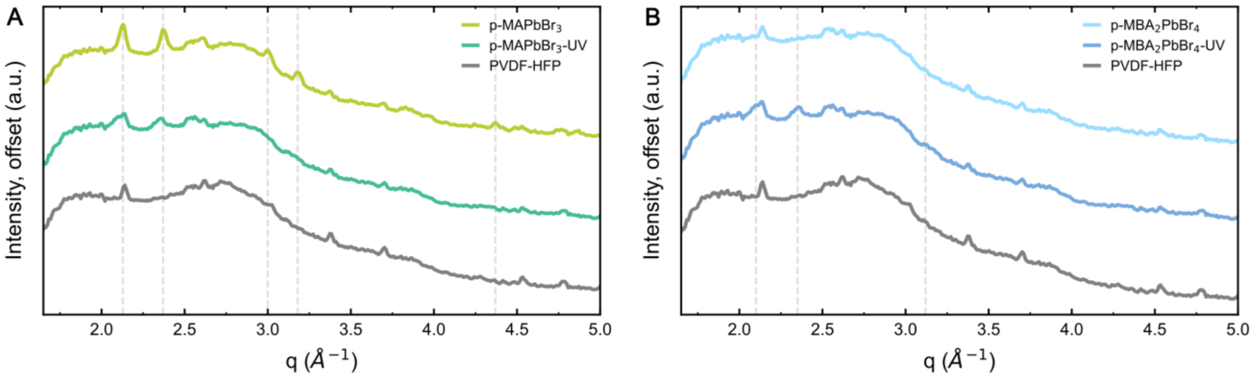

Figure S2. Wide-angle X-ray scattering (WAXS). (A) WAXS spectra of pristine and UV exposed $MAPbBr_3$ and **(B)** MBA_2PbBr_4 polymer composite films with the PVDF-HFP spectrum as reference. The dashed gray lines are intended as guides to the eye to indicate the position of the peaks changing upon UV exposure.

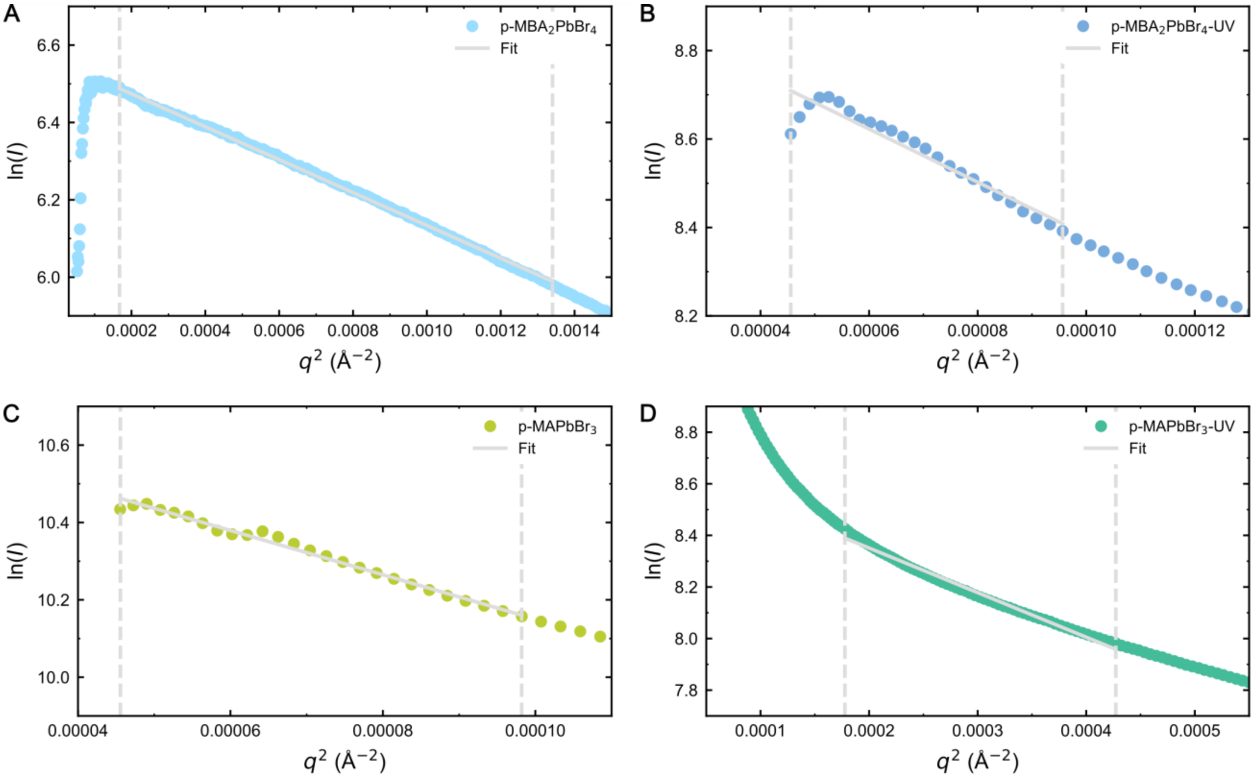

Figure S3. Guinier plots. (A) Guinier fit (in gray) of small angle X-ray scattering (SAXS) Measurements of pristine and **(B)** UV exposed MBA_2PbBr_4, as well as **(C)** pristine and **(D)** UV exposed $MAPbBr_3$ perovskite-polymer composites to estimate the radius of gyration with q as the scattering vector and the I the scattered intensity. Gray dashed lines indicate the minimum and maximum q-values used for the fit. The radii of gyration are 3.6 nm (p-MBA_2PbBr_4), 13.4 nm (p-MBA_2PbBr_4-UV), 13.1 nm (p-$MAPbBr_3$) and 7.2 nm (p-$MAPbBr_3$-UV), respectively. Note that the increase in intensity at low q seen for $MAPbBr_3$-UV is characteristic for aggregates forming. This is likely due to the UV-radiation damaging and breaking apart individual nanocrystals, which then remain in close proximity. Therefore, the resulting size estimate for $MAPbBr_3$-UV is likely less reliable compared to the other samples.

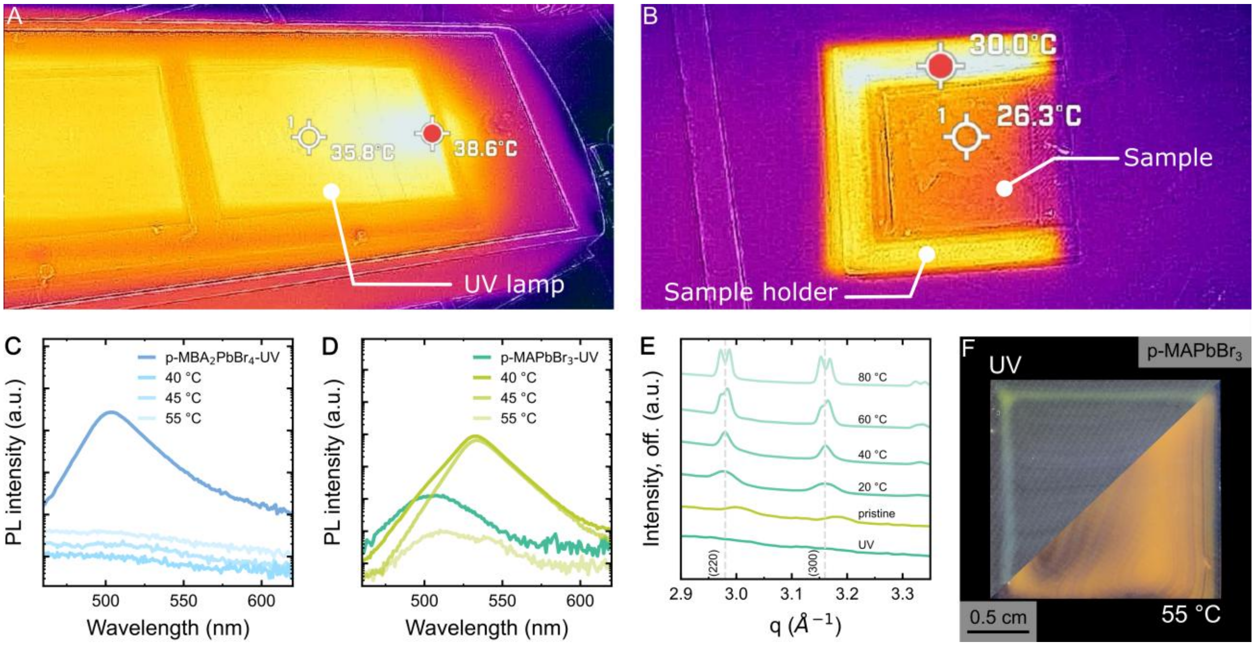

Figure S4. Heat exposure. (A) Thermal image of the used UV lamp and **(B)** the sample inside the sample holder immediately after UV-exposure for 7 min. The red red dot indicates the hottest point in the image (38.6 and 30.0 °C, respectively). **(C)** PL spectra of MBA_2PbBr4 **(D)** $MAPbBr_3$ polymer composites exposed to 40-55 °C with UV treated films as reference. **(E)** XRD patterns of heat exposed $MAPbBr_3$ films as well as pristine and UV-treated samples. **(F)** Image of $MAPbBr_3$ films subjected to either 55 °C (bottom right) or UV light (top left) during fabrication to illustrate the optical difference between UV and heat induced PL quenching.

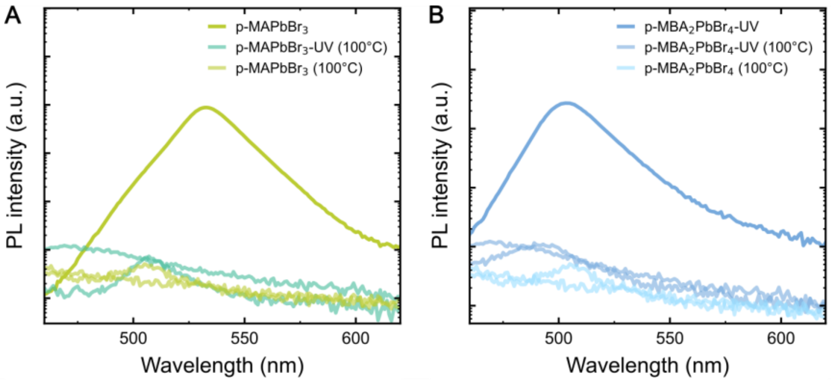

Figure S5. Film annealing. PL spectra of **(A)** p-$MAPbBr_3$ and **(B)** p-MBA_2PbBr_4 films that were either processes using the vacuum drying step or annealed at 100 °C for 10 min.

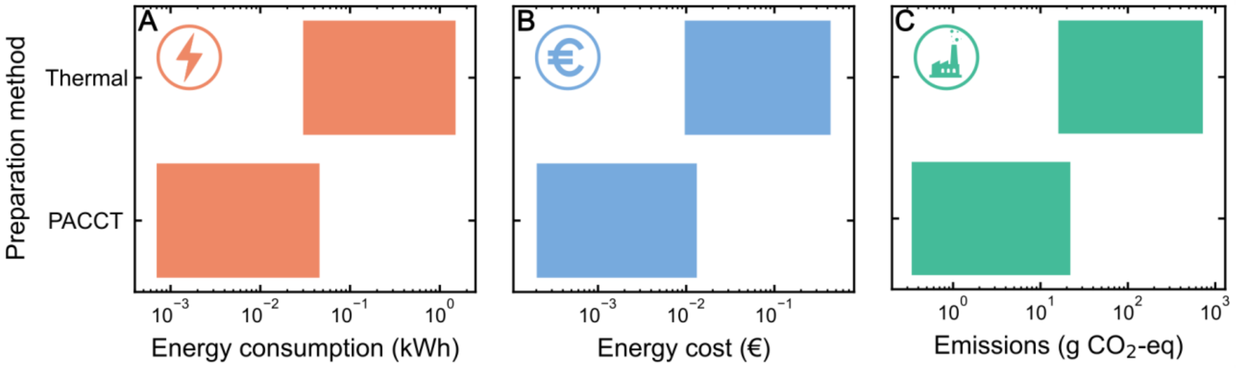

Figure S6. Comparison of preparation methods. (A) Estimation of energy consumption, associated with **(B)** monetary cost as well as **(C)** greenhouse gas emissions of conventional perovskite crystallization methods relying on elevated temperatures (thermal) and the photochemically assisted crystallization control technique (PACCT) presented in this work. The values used for the calculation are listed in Table S2.

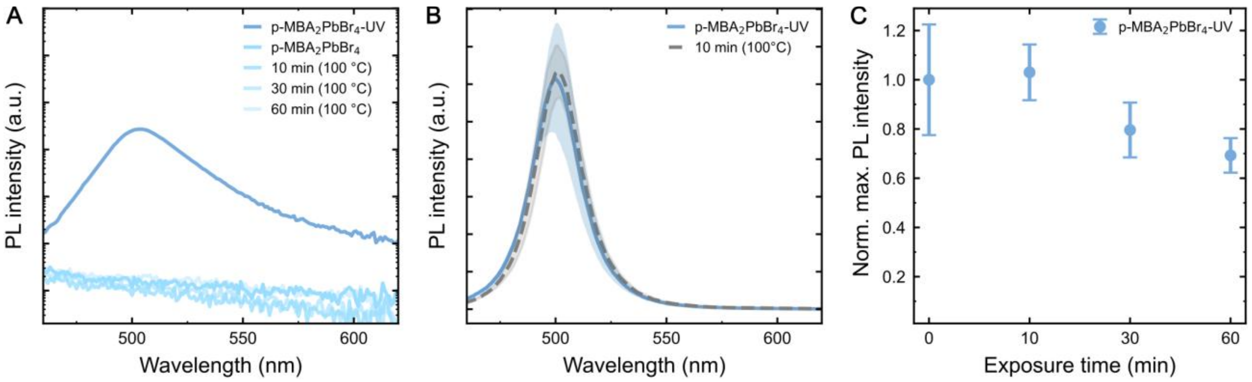

Figure S7. Heat stability. (A) PL spectra of p-MBA_2PbBr_4 films after continuous annealing for 10-60 minutes at 100 °C, demonstrating that they do not become emissive. P-MBA_2PbBr_4-UV is displayed as a reference. **(B)** PL spectra and **(C)** normalized maximum PL intensity of p-MBA_2PbBr_4-UV films after being exposed to 100 °C for different times. Error bands and error bars correspond to the standard deviation of four samples.

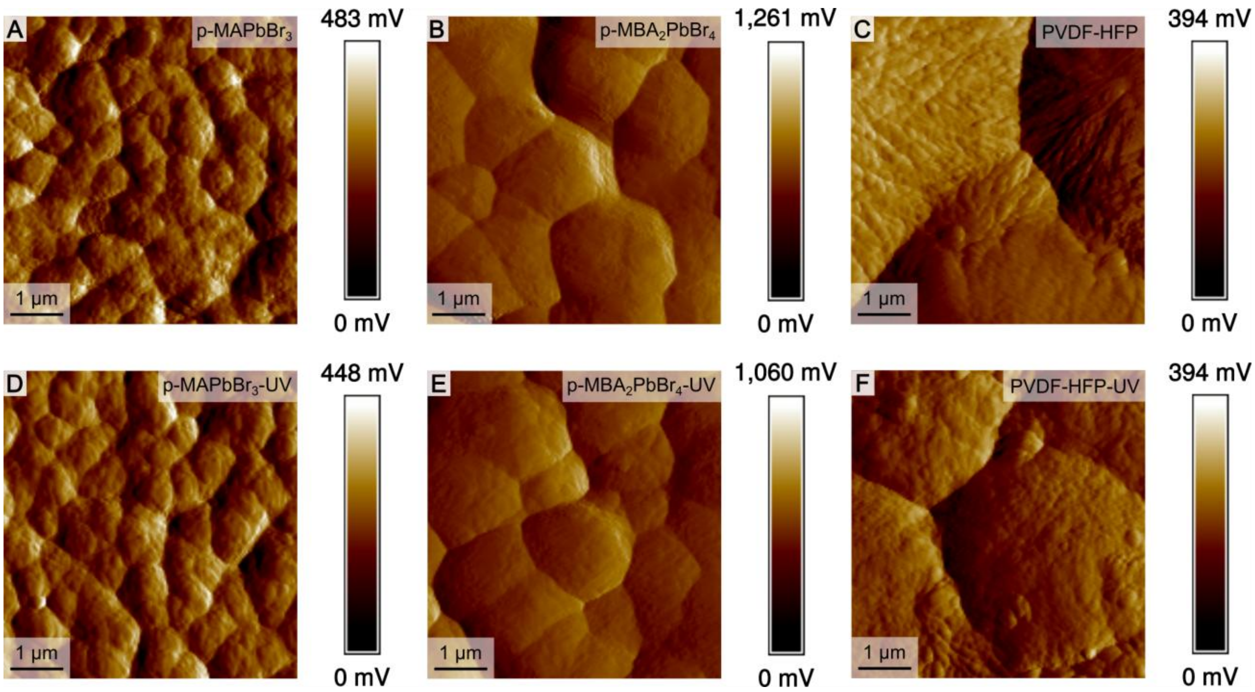

Figure S8. Atomic force microscopy (AFM). AFM images of **(A-C)** pristine and **(D-F)** UV exposed $MAPbBr_3$, MBA_2PbBr_4 and PVDF-HFP films.

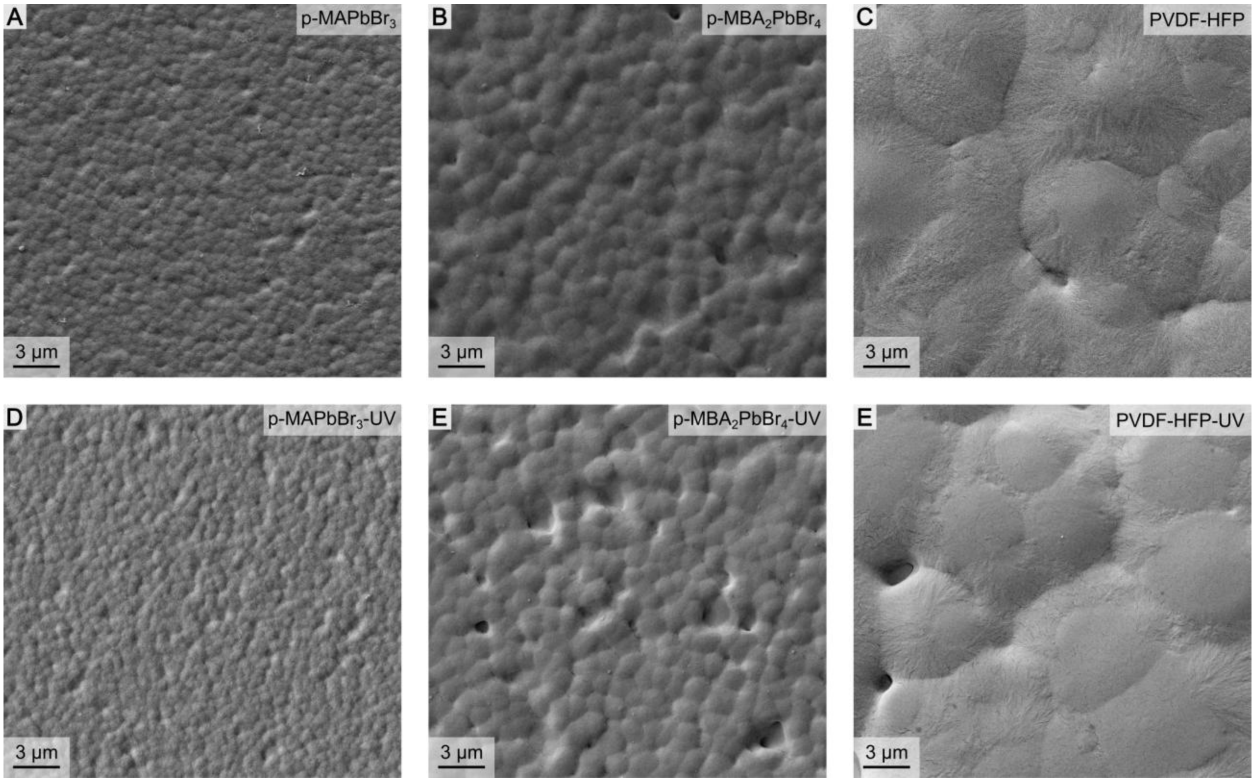

Figure S9. Scanning electron microscopy (SEM). SEM images of **(A-C)** pristine and **(D-F)** UV exposed $MAPbBr_3$ and MBA_2PbBr_4 perovskite-polymer composites as well as PVDF-HFP films.

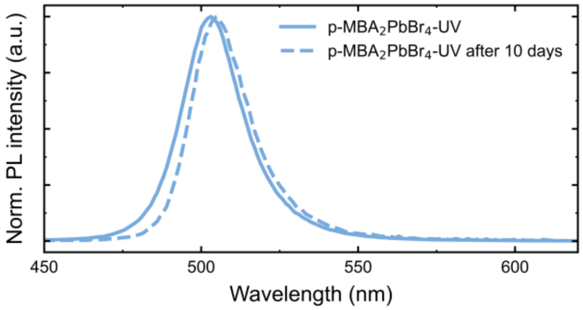

Figure S10. Water Stability. PL Spectra of p-MBA_2PbBr_4-UV films before and after 10 days of full submersion in water.

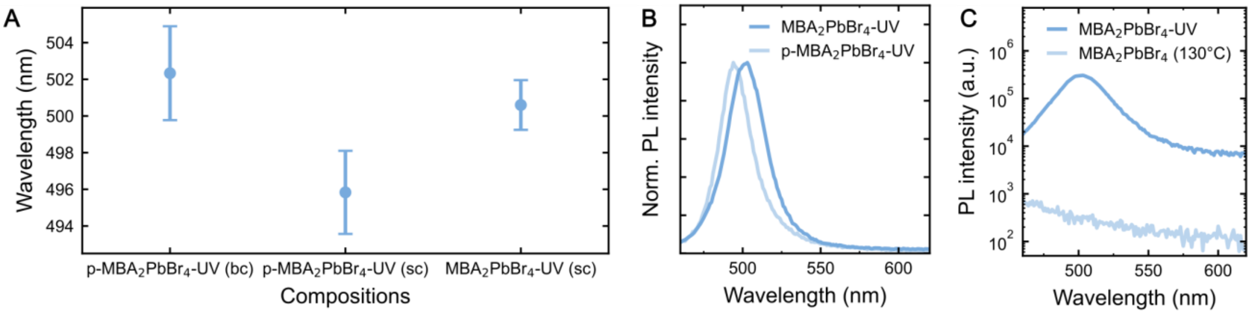

Figure S11. Polymer vs. polymer-free films. (A) Maximum PL peak position and **(B)** comparison of normalized PL spectra of UV exposed MBA_2PbBr_4 films fabricated with and without PVDF-HFP. The abbreviations bc and sc stand for blade coated and spin coated samples respectively. Error bars correspond to 6 (p-MBA_2PbBr_4 (bc)), 5 (p-MBA_2PbBr_4 (sc)) and 4 (MBA_2PbBr_4 (sc)) samples. Note that the differences in PL peak position between the different preparation mechanisms are most likely related to the different drying times: As the spin coated films are thinner, the polymer dries faster and the PNCs have thus less time to form. This further reduces their size, shifting their maximum PL peak position to smaller wavelengths due to quantum confinement. **(C)** PL spectra of polymer-free MBA_2PbBr_4 fabricated by either using PACCT or thermal annealing at 130°C for 10 min.

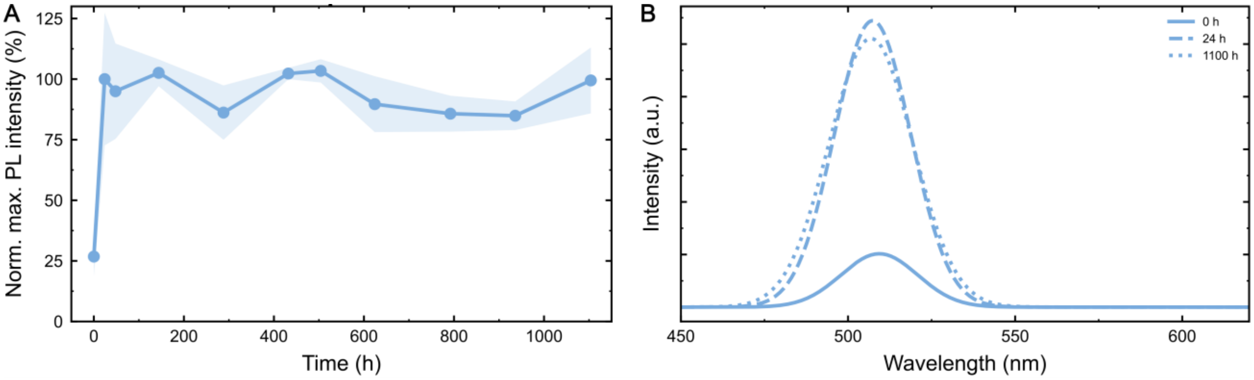

Figure S12. Humidity exposure. (A) Prolonged exposure of MBA_2PbBr_4 polymer-free, UV treated thin films to a controlled humid atmosphere (80 % RH, 23 °C). Maximum PL intensities

are normalized to intensities measured after 24 h. The data points mark the mean intensity of 4 samples, the blue area the standard deviation. Note that the initial measurements (0 h) were performed immediately after processing and so the samples did not yet have time to fully form. As they are left for several hours in ambient conditions, the PL continuously improves as the amine is reprotonated by residual HBr or ambient moisture (as described in the Supplementary Note). **(B)** Comparison of the PL spectra of the same samples at different times.

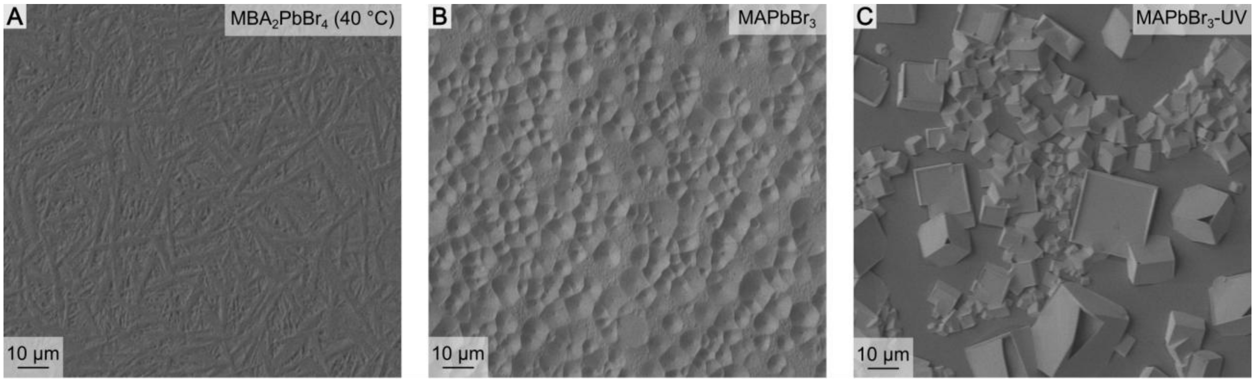

Figure S13. Polymer-free films. SEM images of **(A)** a pristine (no UV) polymer-free MBA_2PbBr_4 film annealed at 40 °C instead of drying in a low pressure environment and **(B)** a pristine as well as a **(C)** UV-exposed $MAPbBr_3$ film.

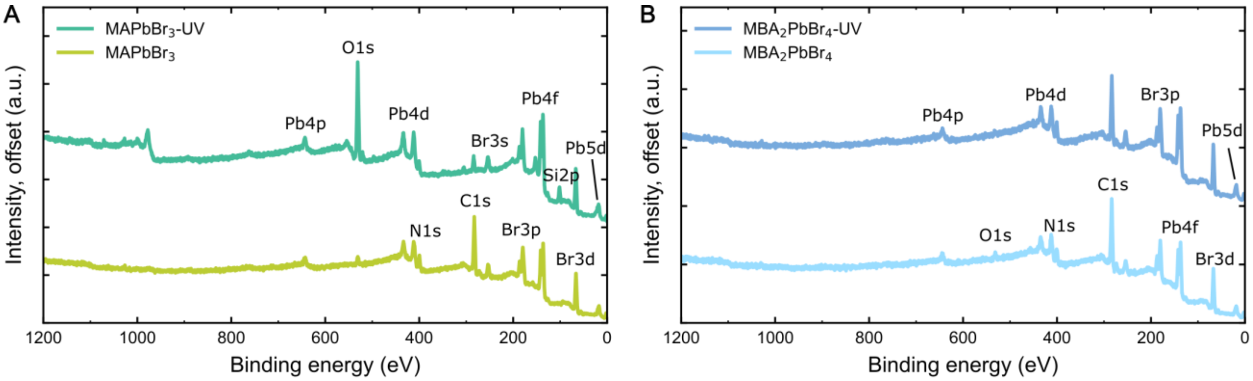

Figure S14. X-ray photoelectron spectroscopy. XPS measurements of polymer-free thin films of pristine and UV-exposed **(A)** $MAPbBr_3$ and **(B)** MBA_2PbBr_4. Note that Si is detected in $MAPbBr_3$-UV films likely due to the degraded films no longer achieving full surface coverage, therefore revealing the glass substrate underneath.

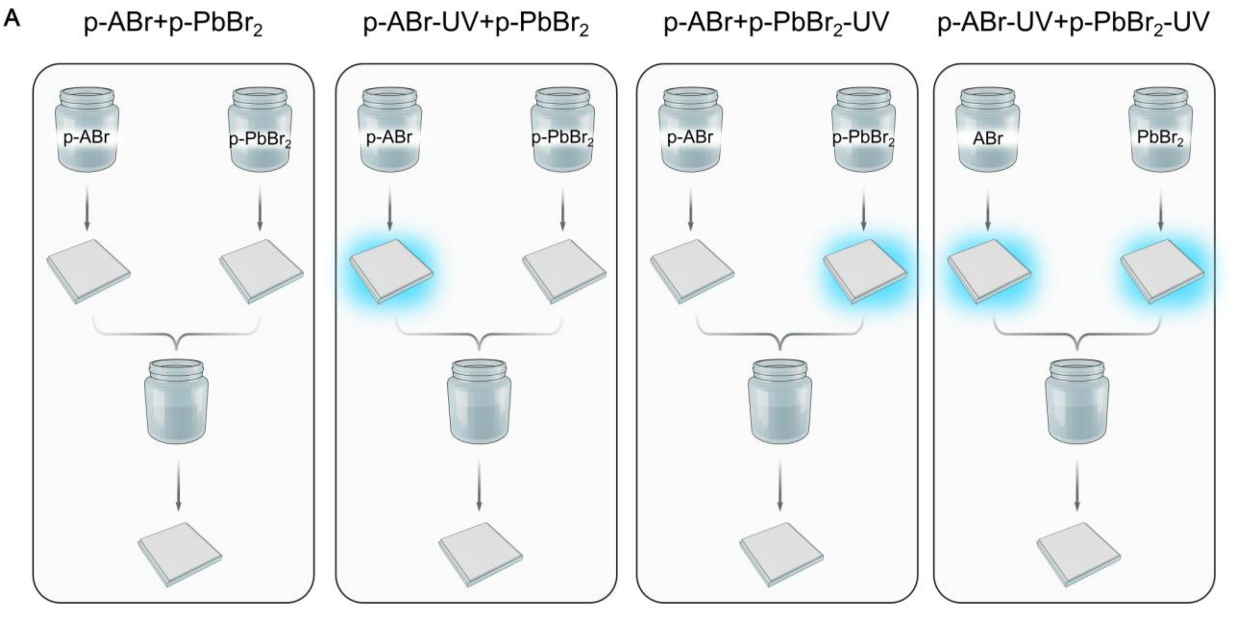

Figure S15. Selective UV-exposure experiment. (A) Illustration of the experiment: Precursor solutions containing PVDF-HFP, DMF and either ABr (A denoting MA or MBA) or $PbBr_2$ were deposited onto glass substrates. A bluish hue around the sample indicates its exposure to UV illumination. New precursor solutions were fabricated by peeling off, and subsequently redissolving these films in DMF and mixing them, followed by blade-coating on a glass substrate. A_xPbBr_y refers to either $MAPbBr_3$ or MBA_2PbBr_4.

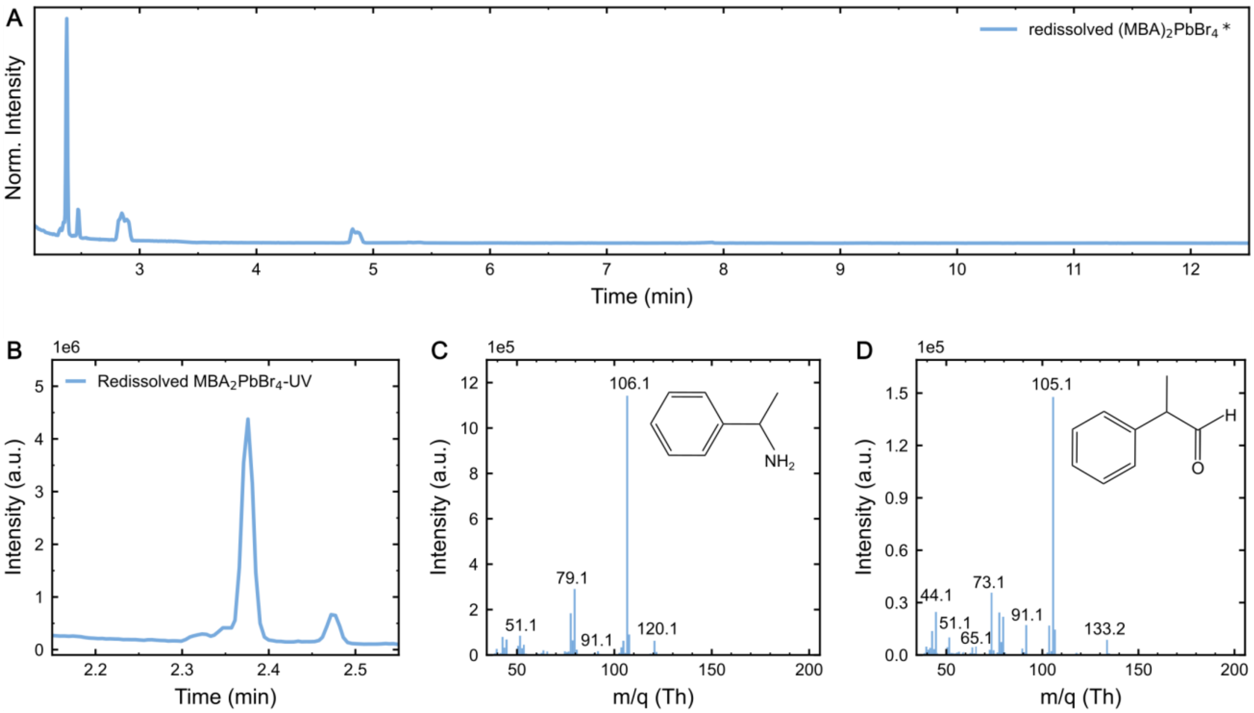

Figure S16. Chemical analysis. (A) Full and **(B)** cropped GC measurement of a redissolved polymer-free, UV-treated MBA_2PbBr_4 film. **(C)** MS profile of peaks appearing at 2.37 min (methylbenzylamine) and **(D)** 2.47 min (2-phenylpropanal) in (B).

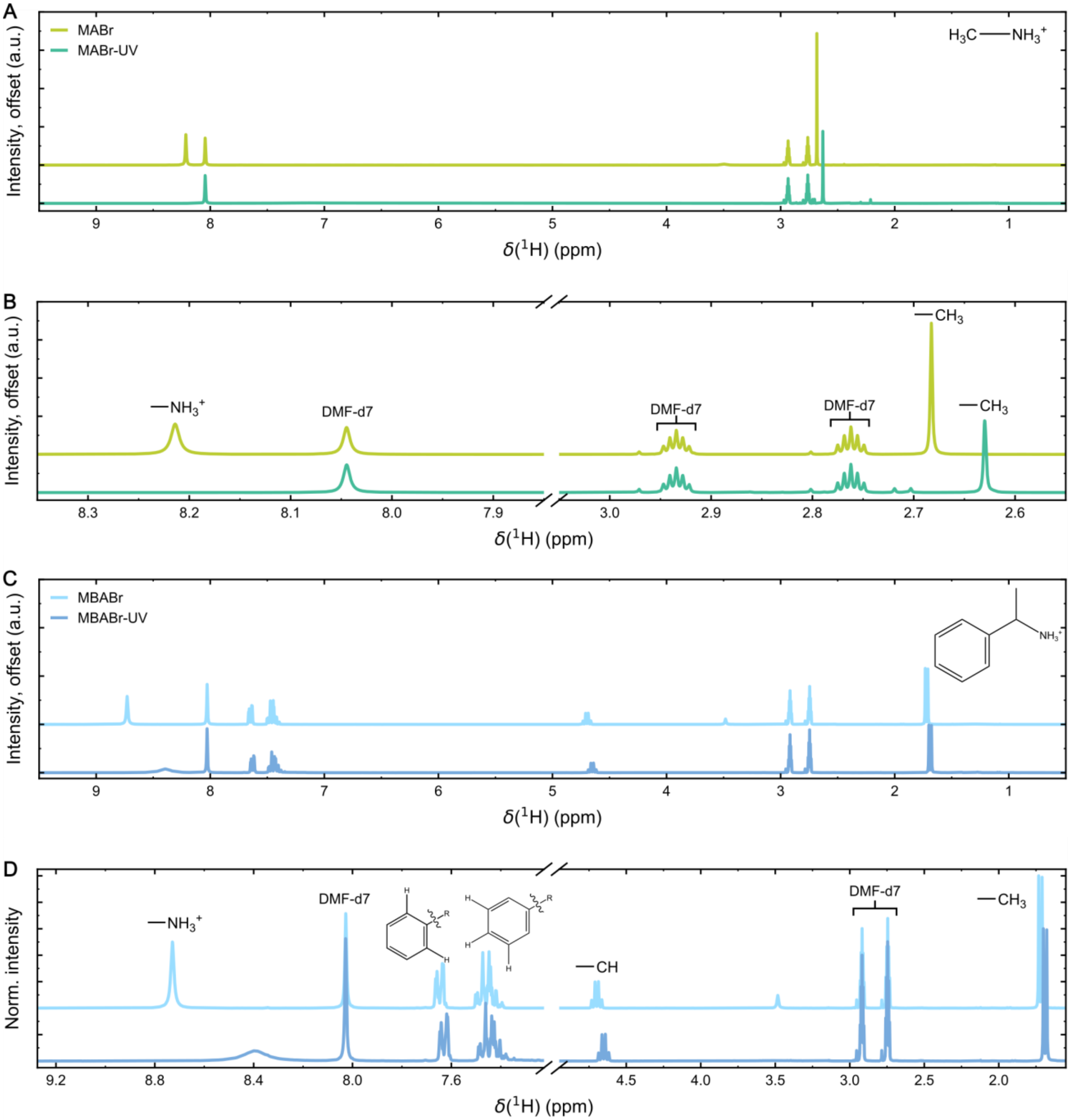

Figure S17. Nuclear magnetic resonance (NMR). (A) NMR spectra of pristine and UV exposed polymer-free MABr solution in full and **(B)** partially magnified for better visibility. **(C)** NMR spectra of pristine and UV exposed polymer-free MBABr solution in full and **(D)** partially magnified for better visibility.

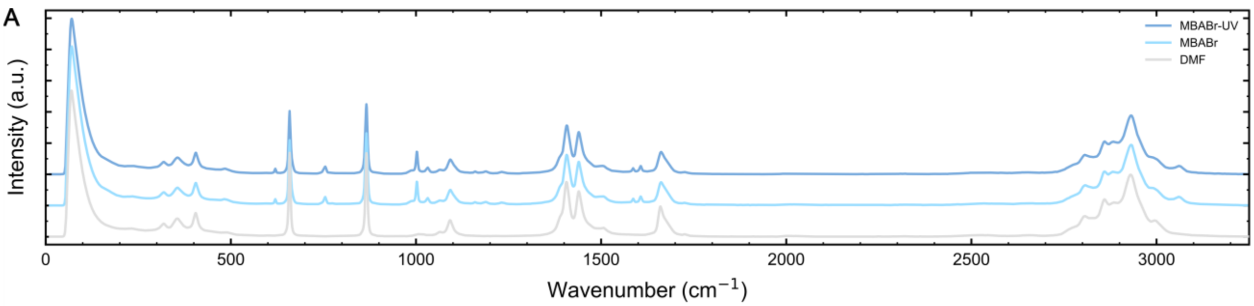

Figure S18. Raman spectroscopy. (A) Raman spectra of polymer-free pristine and UV exposed MBABr solution with DMF as reference.

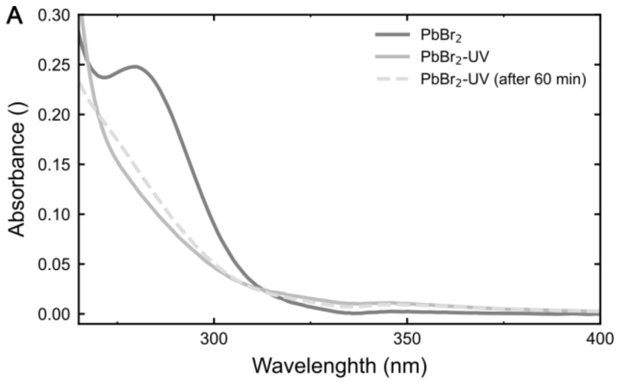

Figure S19. Lead bromide. (A) Absorbance of pristine and UV treated (exposure time of 30 min) diluted $PbBr_2$ (10^{-4} M) dissolved in DMF. After UV exposure, the sample was left to rest for 60 min in a dark environment and subsequently remeasured to determine if the observed changes are reversible.

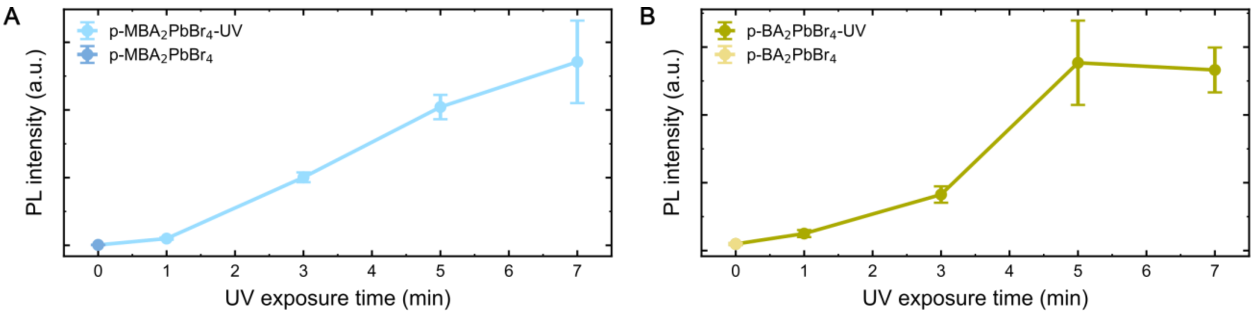

Figure S20. Steric hindrance. PL intensities of **(A)** MA2PbBr4 and **(B)** BA_2PbBr_4 after UV illumination from 0-7 min during the first UV exposure step. The errorbars represent 3 samples. The continuous line serves as a guide to the eye.

Supplementary Tables

Table S*2*: PLQY. Average and maximum PLQY of p-MBA_2PbBr_4-UV prepared using varying molar ratios of MBABr:$PbBr_2$. Errors represent the standard deviations of three samples.

**MBABr:$PbBr_2$**	**PLQY (%)**	**Max. PLQY**
0.5:1	27.0 ± 2.0	28.6
0.75:1	27.7 ± 0.3	28.0
1:1	25.8 ± 0.2	25.9
1.25:1	19.0 ± 1.0	20.0
1.6:1	14.0 ± 5.0	15.7

Table S*3*: Estimates. Estimates of the energy consumption from different fabrication methods, as well as the resulting costs and greenhouse gas emissions. The price was calculated using the average energy price in the European Union for household consumers in 2024 of 28.89 EURc/kWh (including all taxes and levies) [8]. Greenhouse gas emissions were estimated based on the average worldwide emission from electricity of 481 gCO_2 kWh^{-1} [9]. For the PACCT method, the upper limit was calculated assuming 14 min UV illumination combined with 15 min vacuum treatment, while the lower limit includes just 7 min UV exposure.

**Method**	**Processing tool**	**Power rating** (W)	**Duration** (min)	**Energy consumption** (kWh)	**Energy cost** (EURc)	**Emission from electricity** (gCO2/kWh)
Thermal	Hot plate	200-1500	10 - 60	0.03 - 1.5	0.86 - 43.3	48 - 722
PACCT	UV lamp	6	7 - 14	$(7 - 14)\cdot 10^{-4}$	0.02 - 0.04	0.34 - 0.67
	Vac. pump	180	7 - 15	0.02 - 0.05	0.6 - 1.3	10.1 - 21.6

Table S*4*: XPS. Composition of polymer-free pristine and UV exposed $MAPbBr_3$ and MBA_2PbBr_4 films as measured using XPS.

**Element**	**$MAPbBr_3$ (Atom%)**	**$MAPbBr_3$-UV (Atom%)**	**MBA_2PbBr_4 (Atom%)**	**MBA_2PbBr_4-UV (Atom%)**
C	61.3	18.6	65.7	64.6
N	8.2	7.0	10.6	11
O	4.3	44.6	4.6	1.6
Br	23.5	26.0	16.0	18.6
Pb	2.7	3.9	3.3	4.2

Supplementary References

[1] B. Hailegnaw *et al.*, "Flexible quasi-2D perovskite solar cells with high specific power and improved stability for energy-autonomous drones," *Nat. Energy*, vol. 9, no. 6, pp. 677–690, 2024, doi: 10.1038/s41560-024-01500-2.

[2] M. Jung, S. G. Ji, G. Kim, and S. Il Seok, "Perovskite precursor solution chemistry: From fundamentals to photovoltaic applications," *Chem. Soc. Rev.*, vol. 48, no. 7, pp. 2011–2038, 2019, doi: 10.1039/c8cs00656c.

[3] Q. Feng *et al.*, "Governing PbI6 octahedral frameworks for high-stability perovskite solar modules," *Energy Environ. Sci.*, vol. 15, no. 10, pp. 4404–4413, 2022, doi: 10.1039/d2ee02162e.

[4] Y.-R. Luo, *Comprehensive Handbook of Chemical Bond Energies*. CRC press, 2007.

[5] L. Lu *et al.*, "Interaction of the Cation and Vacancy in Hybrid Perovskites Induced by Light Illumination," *ACS Appl. Mater. Interfaces*, vol. 12, no. 37, pp. 42369–42377, 2020, doi: 10.1021/acsami.0c11696.

[6] S. Wang, Y. Jiang, E. J. Juarez-Perez, L. K. Ono, and Y. Qi, "Accelerated degradation of methylammonium lead iodide perovskites induced by exposure to iodine vapour," *Nat. Energy*, vol. 2, no. 1, pp. 1–8, 2017, doi: 10.1038/nenergy.2016.195.

[7] D. P. McMeekin *et al.*, "Crystallization Kinetics and Morphology Control of Formamidinium–Cesium Mixed-Cation Lead Mixed-Halide Perovskite via Tunability of the Colloidal Precursor Solution," *Adv. Mater.*, vol. 29, no. 29, 2017, doi: 10.1002/adma.201607039.

[8] "Electricity prices for household consumers - bi-annual data (from 2007 onwards)," *Eurostat*. https://doi.org/10.2908/NRG_PC_204

[9] International Energy Institute (IEI), "Statistical Review of World Energy 2023," *BP Energy Outlook 2023*, vol. 70, pp. 8–20, 2023.
